# Effectiveness of Internet-Based Interventions on Glycemic Control in Patients With Type 2 Diabetes: Meta-Analysis of Randomized Controlled Trials

**DOI:** 10.2196/jmir.9133

**Published:** 2018-05-07

**Authors:** Ying Shen, Fengbin Wang, Xing Zhang, Xiaorou Zhu, Qiudan Sun, Edwin Fisher, Xinying Sun

**Affiliations:** ^1^ School of Public Health Peking University Health Science Center Beijing China; ^2^ Department of Health and Education Beijing Municipal Commission of Health and Family Planning Beijing China; ^3^ Institute for Medical Humanities Peking University Health Science Center Beijing China; ^4^ Gillings School of Global Public Health University of North Carolina North Carolina, NC United States

**Keywords:** internet, type 2 diabetes mellitus, HbA_1c_, randomized controlled trial, meta-analysis

## Abstract

**Background:**

The popularity of internet as an area of research has grown manifold over the years. Given its rapid development and increasing coverage worldwide, internet-based interventions seem to offer a promising option to ameliorate huge burdens brought by type 2 diabetes mellitus. However, studies conducted by different researchers have provided contradictory results on the effect of internet-based interventions in glycemic control.

**Objective:**

This meta-analysis aims to summarize currently available evidence and evaluate the overall impact of internet-based interventions on glycemic management of type 2 diabetic patients.

**Methods:**

A systematic literature search was performed in PubMed, ScienceDirect, and Web of Science. Randomized controlled trials that used glycosylated hemoglobin values as the outcome measure of glycemic control were considered. Risk of bias and publication bias were evaluated.

**Results:**

Of the 492 studies, 35 were included in meta-analysis, and results indicated that the weighted mean difference (WMD) between usual care and internet-based interventions at endpoint was –0.426% (95% CI –0.540 to –0.312; *P*<.001). Subgroup analyses revealed that intervention duration ≤3 months yielded optimal performance (WMD –0.51%; 95% CI –0.71 to –0.31; *P*<.001). Combined mobile and website interventions were substantially superior to solely Web-based and mobile-based interventions in glycemic control (combined WMD –0.77%, 95% CI –1.07 to –0.47; *P*<.001; Web only: WMD –0.48%; 95% CI –0.71 to –0.24, *P*<.001; mobile only WMD –0.31%, 95% CI –0.49 to –0.14; *P*<.001). Furthermore, the effect of interventions with automated feedbacks was similar to those with manual feedbacks, and studies with internet-based educational contents were more effective in glycemic control. The assessment revealed a low risk of bias.

**Conclusions:**

In conclusion, utilization of internet-based intervention is beneficial for patients with type 2 diabetes mellitus, and taking full advantage of this type of intervention may substantially reduce the incidence of complications and improve quality of life.

**Trial Registration:**

International Prospective Register of Systematic Reviews (PROSPERO): CRD42017058032; https://www.crd.york.ac.uk/PROSPERO/display_record.php?RecordID=58032 (Archived by WebCite at http://www.webcitation.org/6yY7eQNHr)

## Introduction

With increasing prevalence and serious chronic and acute complications, diabetes has brought enormous burden to people’s living and production. Glycemic control is vital in disease management as hyperglycemia and hypoglycemia can both lead to serious clinical consequences. However, in some areas, health care providers can only obtain patients’ information on glycemic control and lifestyle during their consultation with a doctor, which is probably infrequent because of various reasons, such as patients’ economic issues, educational levels, health awareness, and remote distance to health care centers. Hence, information obtained by doctors cannot comprehensively reflect patients’ health status and may lead to suboptimal health decisions [1]. Therefore, most of the impetus of disease management is on the patients as diabetes is a life-course chronic condition, and health care providers cannot possibly accompany patients all the time [2]. As a result, to achieve wide-scale promotion of diabetes self-management, it is essential to explore an efficient, easily available, and cost-effective approach to bridge geographical and economic gaps and reach broader populations. Given the rapid development of new technology and penetration of internet in people’s daily life, integrating diabetic inventions into existing technologies may have important implications and accord with clinical guidelines as they have widely referred to the significance of self-management and patients’ education in diabetes control [3-5]. In this aspect, internet-based interventions perfectly meet the requirement, as they can not only easily reach the global population but also serve as a medium of mass information exchange [6].

Currently, many studies have explored the efficacy of using internet-based interventions in diabetes management. However, there is not a universally acknowledged verdict on whether internet-based interventions could make a significant difference in patients’ glycemic level. Some research reported that compared with usual care, internet-based interventions could significantly reduce glycated hemoglobin (HbA_1c_) levels in patients with type 2 diabetes mellitus (T2DM) [7-11], whereas others reported otherwise [12-16]. Therefore, to comprehensively evaluate the overall impact of internet-based interventions, a meta-analysis is needed to achieve the pooled result. Furthermore, to our best knowledge, although there were some meta-analyses studying the effect of internet-based interventions on diabetes management, most of them just looked at a single form such as mobile apps and few conducted a comprehensive comparison between different forms of internet-based interventions. As a result, by analyzing relevant randomized controlled trials (RCTs) on this issue, this meta-analysis aims to evaluate the feasibility and efficacy of internet-based interventions on diabetes management and hopes to discover the most effective model of intervention that will benefit as many patients as possible.

This meta-analysis was registered at the international prospective register of systematic reviews (no. CRD42017058032).

## Methods

### Data Sources and Searches

For this meta-analysis, we have conducted a search in PubMed, Web of Science, and ScienceDirect to identify studies relevant to this topic from their beginning to January 16, 2017. Keywords used in this search were “T2DM,” “type 2 diabetes,” “social media,” “e-learning,” “new media,” “m-health,” “mHealth,” “internet-based,” “web-based,” “twitter,” “eHealth,” “e-health,” “Facebook,” “computer-delivered,” “social web,” “social software,” “online case-based learning,” “mobile learning,” “digital game-based learning,” “serious games,” “wearable devices,” “mobile app,” “mobile application,” and “smart phone-based.” To achieve a searching scope that was broad enough, the above-mentioned keywords were not enclosed. In PubMed, we searched in all fields and found 291 papers. In both of Web of Science and ScienceDirect, we searched in title, abstracts and keywords and retrieved 228 and 65 papers, respectively. The language of studies was not limited.

To extend our search scope and include more studies in this field, we also manually searched the reference lists of several systematic reviews and meta-analyses that might be related to our own study [6,17-23]. The number of studies identified in this way was 76. Furthermore, in the process of records screening, we found that 1 study was built on a previously published research conducted by the same research group, which examined the efficacy of improvement made to the previous program and evaluated people’s acceptance. As a result, the study of the previous research was also found through the reference list and was included. Therefore, a total of 77 studies were retrieved from the references lists of other research. After the removal of duplicates, 492 records were identified in total. Multimedia Appendix 1 shows details of search syntax.

### Study Selection

Two reviewers, Shen and Wang, independently reviewed abstracts and full-text papers. Study selection was strictly based on uniform inclusion and exclusion criteria. The exclusion reason for each study was recorded, and in the case of disagreement, Sun was consulted, and discussion was held until a consensus was reached. Studies were included if they were RCTs, assessed patients aged older than 18 years with T2DM, compared the effect of internet-based interventions in glycemic management with a control group (CG) of usual care, and reported means and SDs of HbA_1c_ values for intervention group (IG) and CG both at baseline and endpoint or other relevant data from which mean and SD of HbA_1c_ could be calculated. If information on diabetes type was not clear, studies with the mean age of patients older than 30 years were included as there is a high possibility that they have T2DM [24]. Studies were excluded if they were nonrandomized, reviews, protocols, case reports, or commentaries; did not use HbA_1c_ as the outcome measure or there was an incomplete report of HbA_1c_; or included patients with type 1 diabetes or gestational diabetes. Furthermore, we also excluded studies that were not predominantly internet-based or the internet was only used as a supporting or additional tool to intensify the effect of other interventions, as it was difficult to tell whether the study effect was attributable to the internet or the other intervention. Interventions that purely used the mobile phone as a tool to send text messages were also excluded, as this was not internet-based. Moreover, to compare the effect of internet-based interventions with usual care, the CG should use usual care rather than internet-based interventions.

**Figure 1:**
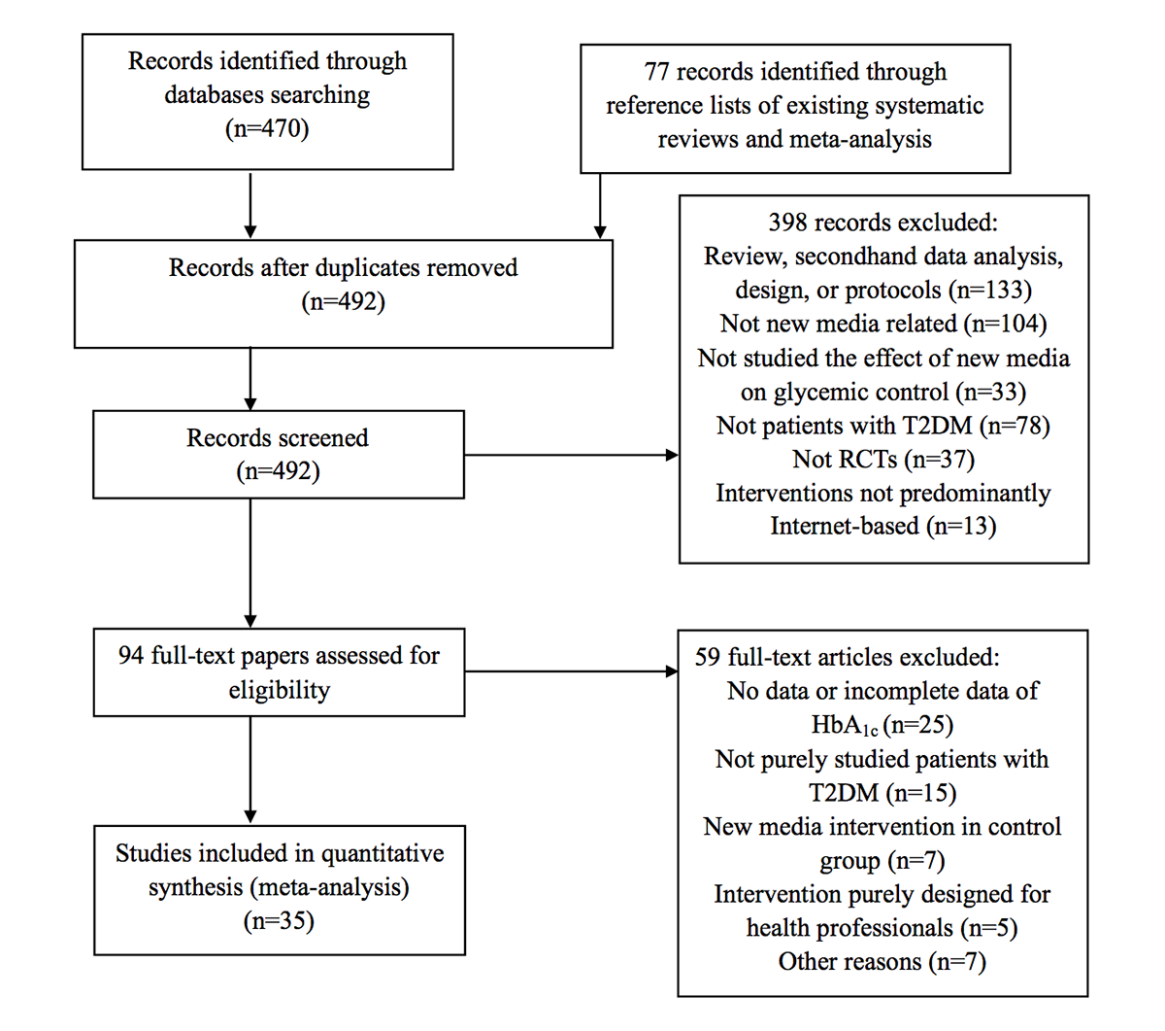
Flowchart of study selection. RCT: randomized controlled trials; T2DM: type 2 diabetes mellitus.

On the basis of retrieved abstracts, we excluded 398 records. We further examined the full texts of the remaining 94 studies, and 59 studies were excluded. A total of 25 studies did not provide data on HbA_1c_ or provided incomplete data on HbA_1c_; 15 studies also studied patients with T1DM; 7 studies used internet-based intervention in CG; interventions in 5 studies were designed for health professionals and still depended on face-to-face interactions; 12 studies reported results of the same 6 interventions, respectively, and thus 6 were excluded; and in 1 study, only 46.1% of patients (n=511) used the internet at least monthly; therefore, the research group decided to send participants paper versions of their medical record and ask them to bring it during the next hospital visit. Therefore, as this research was largely not internet-based, it was also excluded (Figure 1).

### Data Extraction and Quality Assessment

The following data in 35 studies were extracted using a standardized spreadsheet: the first author, year of publication, participants included (CG and IG, respectively), patients’ characteristics (including attrition, completion rate, mean age, body mass index [BMI], and gender ratio), study design, intervention duration, study location, intervention method, HbA_1c_ data (including values at baseline and endpoint), and other useful information. Furthermore, studies included were numbered from 1 to 35. One study had 2 IGs that met the eligibility criteria and was numbered 12a and 12b.

Mean values and SD of HbA_1c_ at baseline and endpoint were extracted separately for IGs and CGs. Some studies only presented changes of HbA_1c_, and in this case, HbA_1c_ values at endpoint were calculated correspondingly based on baseline and changes of values [8,16,25-27]. For studies that provided SE rather than SD, SD value was calculated based on the SE value [12,28-30]. Some studies gave 95% CI of mean HbA_1c_, and SD was derived according to 95% CI [14,25,31]. If neither endpoint SD nor other information that could be used to calculate SD was provided, baseline SD was adopted as endpoint SD [26,32].

Cochrane collaboration’s tool for assessing the risk of bias was adopted to evaluate the quality of studies included. Six domains were assessed, including selection bias, performance bias, detection bias, attrition bias, reporting bias, and other bias.

### Data Synthesis and Analysis

Interstudy heterogeneity was evaluated by the *I*^2^ test. If *I*^2^≤50%, heterogeneity across studies was acceptable, and fixed effect model could be used to achieve the pooled result. If *I*^2^>50%, random effect model would be used, and source of heterogeneity would be explored. Meta-analysis with continuous outcome variables was performed, and weighted mean difference (WMD) was adopted as the effect indicator. If *P*<.05 and 95% CI did not include zero, the point estimate of WMD was considered statistically significant. Publication bias was explored by Begg and Egger tests. Duval and Tweedie’s nonparametric “trim-and-fill” procedure was also performed to further assess the possible effect of publication bias [33,34]. Sensitivity analysis was conducted to assess the stability of studies. Statistical analyses were performed with STATA/SE 14.1 (StataCorp LP, College Station, Texas) and risk of bias was evaluated with Review Manager (RevMan 5.3; Cochrane Collaboration).

## Results

### Characteristics of Studies

This meta-analysis included 35 studies with a pooled dataset of 6475 participants, of which 3338 were allocated to the IG and 3137 to CG [7-12,14,16,25-32,35-53]. The total number of patients in each study ranged from 30 to 1665. The characteristics of eligible studies are presented in Multimedia Appendix 2.

Of the 35 studies included, 11 were conducted in the United States [11,12,26,27,29,32,37,39,40,48,50]; 10 in the Republic of Korea [7,8,28,30,35,36,46,49,52,53]; 2 in Poland [38,45], Italy [16,44], and China [43,47] each; 1 in Japan [42], Finland [25], Turkey [10], Canada [9], Spain [31], Norway [14], England [41], and Congo[51] each. Included studies were published between 2004 and 2016, and the intervention duration ranged from 6 weeks to 5 years. The mean age of the participants ranged from 42.3 to 79.9 years and BMI from 22.8 to 36.9. All studies, except 2, clearly stated that all participants had T2DM. However, in these 2 studies, the mean age of the patients was above 60 years; therefore, it was assumed that the included participants were all patients with T2DM [29,50].

### Characteristics of Interventions

A total of 8 studies used Web-based interventions solely [7,9,10,12,28,38,43], 8 used mobile technology [14,25,31,36,42,45,48,52], and 9 used a combination of both [8,35,39,40,46,47,49,51,53]. In addition, 10 studies used other forms of internet-based interventions, including portable digital assistant, tablet computer, and data transmission equipment only [11,16,26,27,29,30,32,37,41,44]. Moreover, 28 studies clearly stated ways of providing feedbacks, among which 5 used automated algorithm [25,36,39,46,52] and 22 provided feedbacks manually via health care providers [7,9-11,14,27-32, 35,37,40,41,43,44,48-51,53]. In the automated algorithm, patient-specific messages were automatically generated and sent to patients based on the data entered into the system. Manual feedback was accomplished through telephone, videoconferencing, and short message services by cellular phone and the internet. A total of 14 studies provided internet-based education [8,10,27,29,32,37,39-41,43,47,49,50,53], and only 3 studies did not incorporate transmission function of glucose measurements [8,12,47]. A detailed description of intervention characteristics is presented in Multimedia Appendix 3.

Publication bias was detected by Egger test but not by Begg test. Trim-and-fill method was further conducted to account for publication bias. In trim-and-fill method, although the strength of intervention was slightly attenuated, the pooled analysis incorporating the hypothetical studies continued to show a statistically significant positive effect of internet-based interventions (WMD –0.332, 95% CI –0.456 to –0.209; *P*<.001; Multimedia Appendix 4), which suggested that the mean difference of HbA_1c_ between IG and CG was not an artifact of unpublished negative studies, but the effect of internet-based interventions. However, the possibility was not altogether excluded by this method. Detailed results on risk of bias, publication bias, and sensitivity analysis can be found in Multimedia Appendices 4-6. Multimedia Appendix 7 describes the HbA_1c_ value and related data of IG and CG at baseline and endpoint.

### Overall Results of Internet-Based Intervention

**Figure 2:**
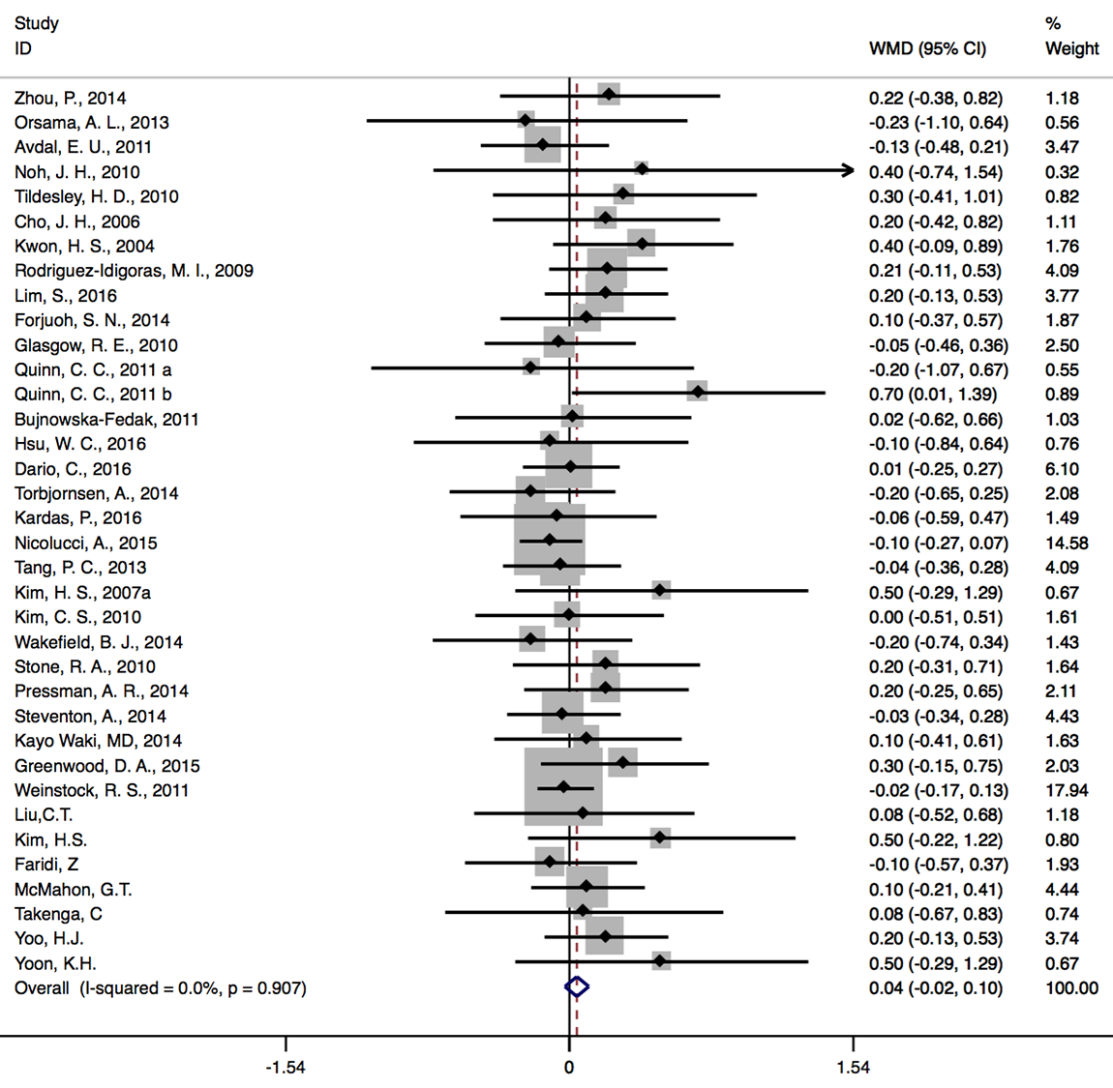
Forest plots of baseline results. WMD: weighted mean difference.

All 35 studies provided mean values and SDs of HbA_1c_ as the outcome measure of internet-based interventions. At baseline, there was no significant difference of HbA_1c_ levels between the CG and IG as 95% CI including zero (Figure 2).

**Figure 3:**
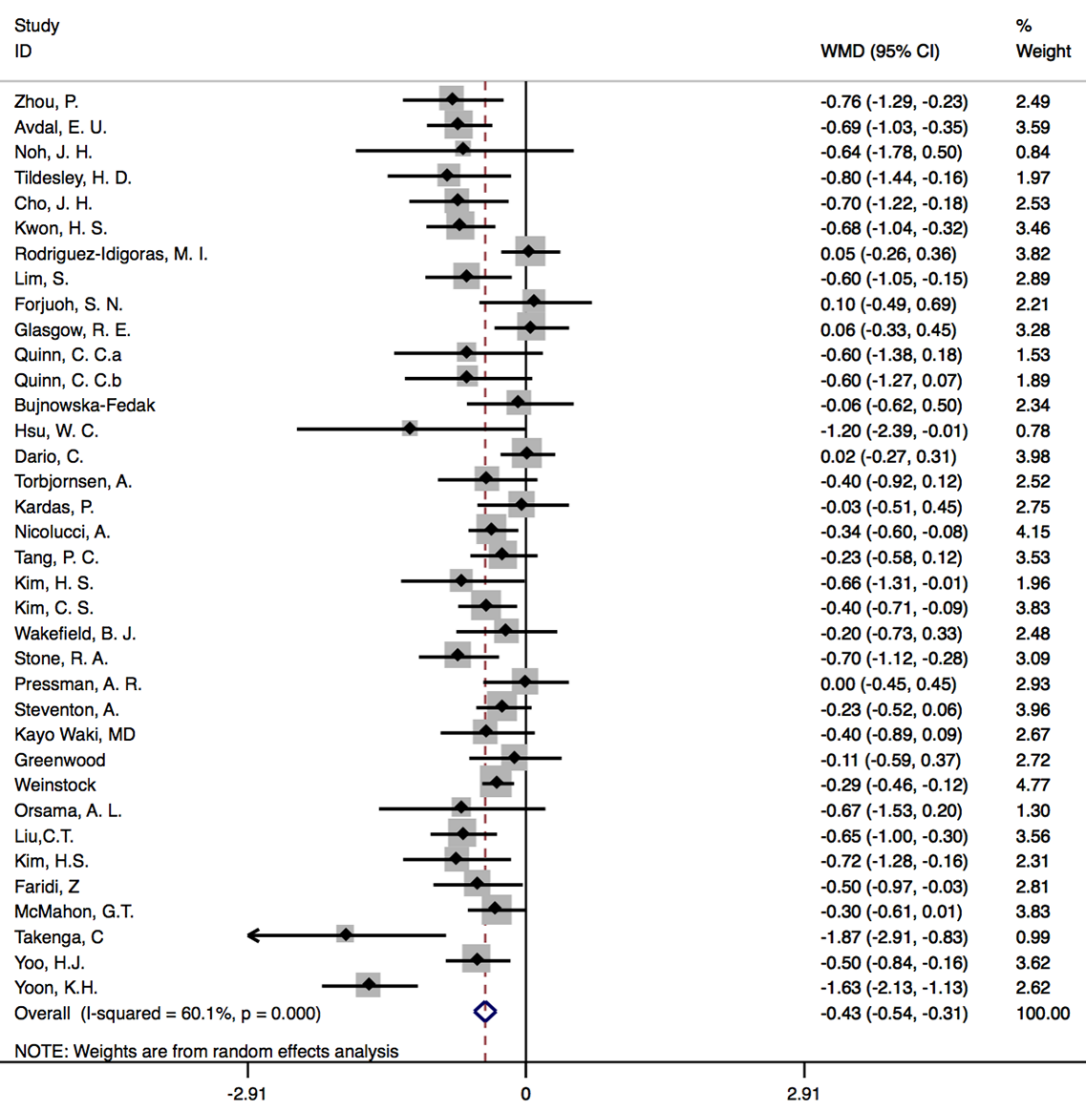
Forest plots of endpoint results. WMD: weighted mean difference.

Measurements made immediately after interventions were used to achieve the overall pooled effect of the internet-based intervention. In one study, 2 IGs met eligibility criteria, and therefore, information was extracted twice and treated as from 2 distinct studies [39]. They were marked as Quinn, C.C., 2011a and Quinn, C.C., 2011b in the analysis. Figure 3 presented the combined results of the 35 studies in this meta-analysis. The result showed that the WMD between CG and IG was –0.426 (95% CI –0.540 to –0.312; *P*<.001), favoring the IG. Heterogeneity was 60.1% across studies, and random effect model was adopted.

### Subgroup Analysis

Subgroup analyses were conducted based on intervention duration, types of internet-based interventions, ways of feedback provision, and whether or not internet-based educational content was provided.

#### Results of Subgroup Analysis Based on Intervention Duration

**Figure 4:**
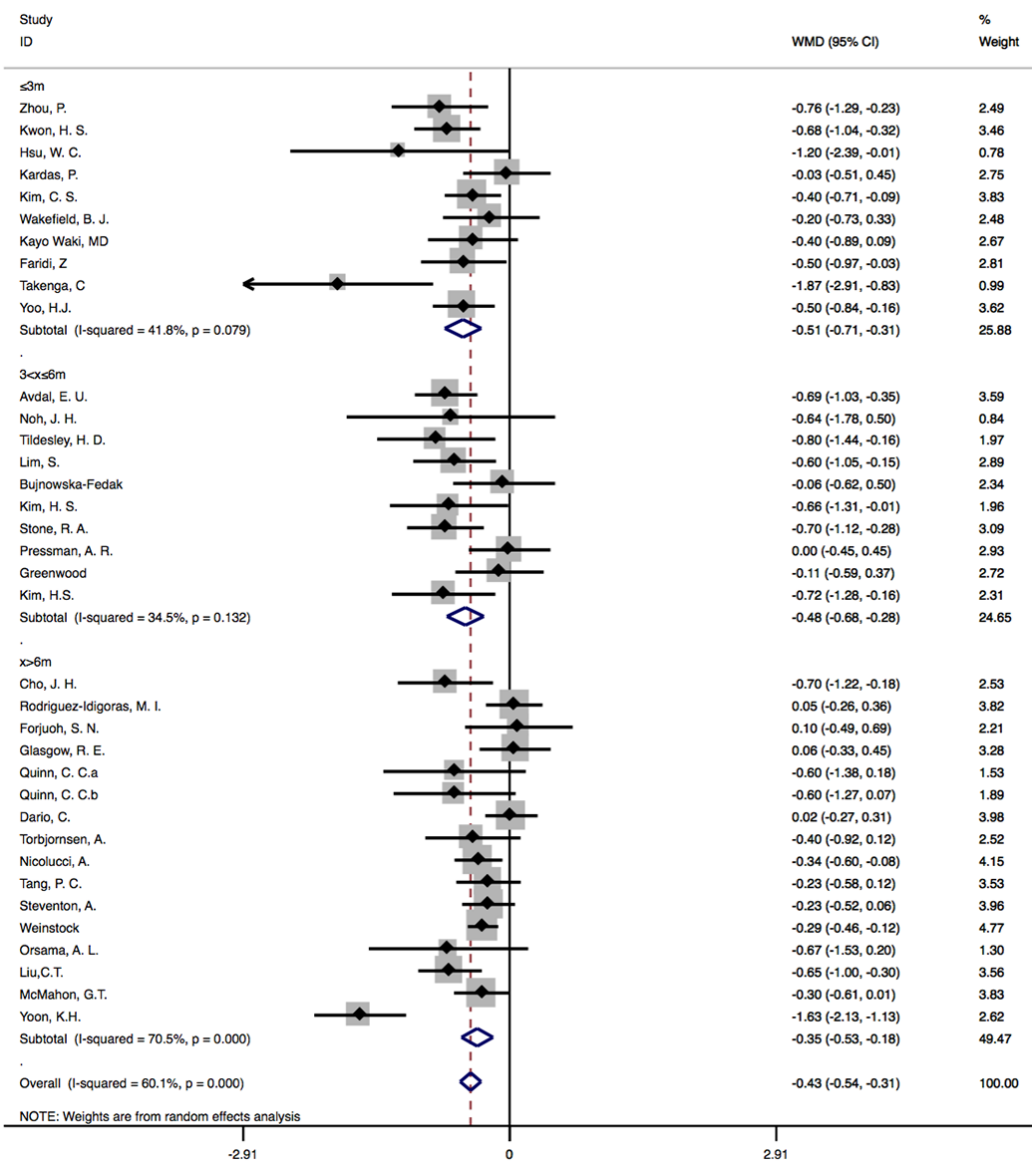
Subgroup analysis based on intervention duration. WMD: weighted mean difference.

As intervention duration in the 35 studies ranged from 12 weeks to 5 years, we divided included research into 3 mutually exclusive groups (x≤3 months: n=10, 3 months<x≤6 months: n=10, and x>6 months: n=16) to perform subgroup analysis. In the group of x≤3 months [11,28,30,36,42,43,45,48,51,52], heterogeneity across studies was 41.8% and the pooled WMD was –0.51% (95% CI –0.71 to –0.31; *P*<.001). Significant difference between IG and CG was also observed in the group of duration >3 months and ≤6 months [8-10,27,32,35,37,38,46,49], with a pooled WMD of –0.48% (95% CI –0.68 to –0.28; *P*<.001). The heterogeneity across study was 34.5%. In the group of duration >6 months [7,12,14,16,25,26,29,31,39-41,44,47,50,53], difference in HbA_1c_ outcomes between IG and CG groups decreased with a WMD of –0.35% (95% CI –0.53 to –0.18; *P*<.001) and heterogeneity increased to 70.5% (Figure 4).

#### Results of Subgroup Analysis Based on the Type of Internet-Based interventions

**Figure 5:**
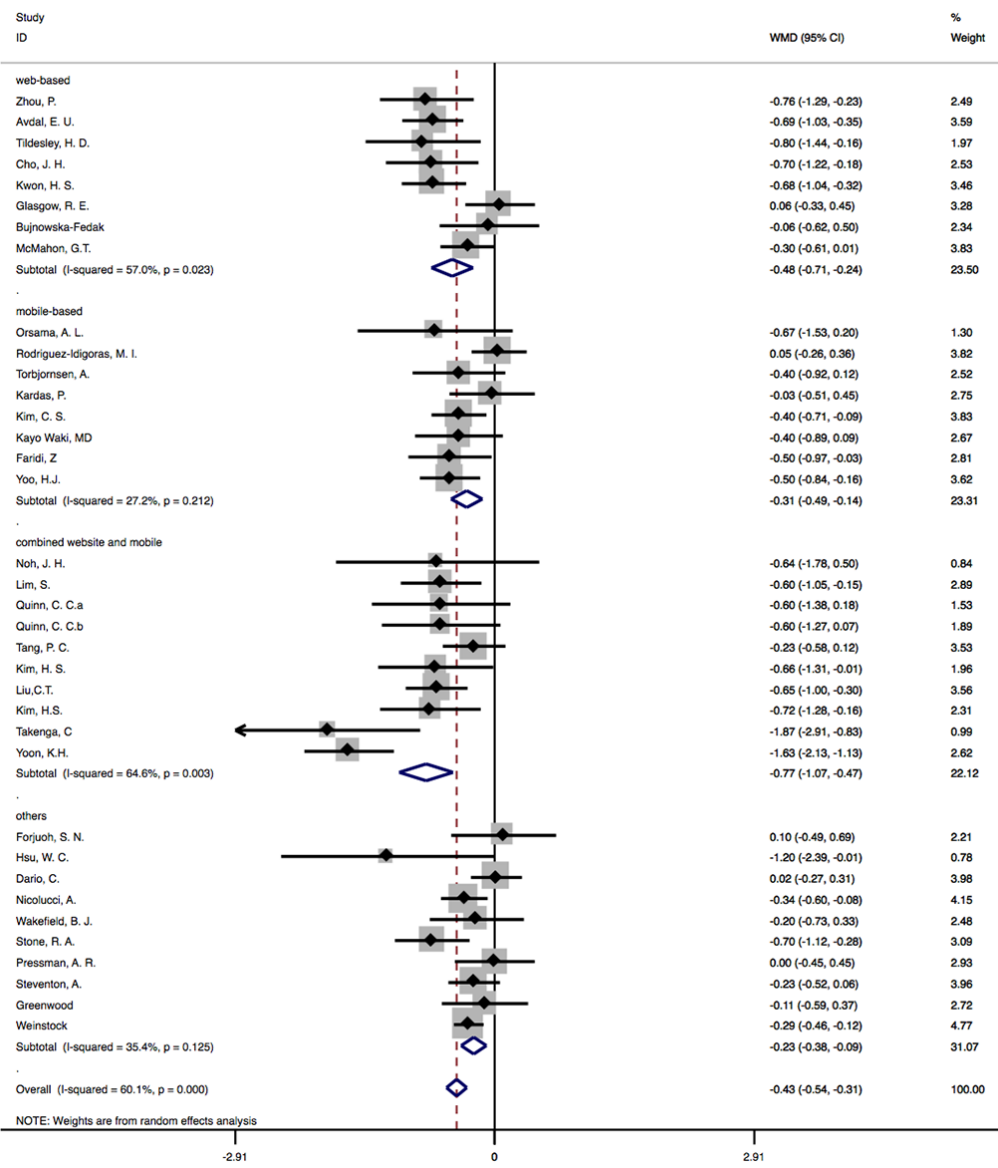
Subgroup analysis based on the type of internet-based interventions. WMD: weighted mean difference.

In this analysis, we divided 35 studies into 4 subgroups based on types of internet-based interventions (Web-based solely: n=8, mobile-based solely: n=8, combined website and mobile technology: n=10, and others: n=10). Forms of intervention that did not fall in the above 3 categories were classified as others. In the group of website-only interventions [7,9,10,12,28,38,43,50], there was statistically significant difference in HbA_1c_ outcomes between IG and CG, and WMD was –0.48% (95% CI –0.71 to –0.24; *P*<.001). However, a moderate heterogeneity of 57% was also observed across studies in this subgroup. In the group of mobile-only interventions [14,25,31,36,42,45,48,52], difference in HbA_1c_ between IG and CG decreased, with a pooled WMD of –0.31% (95% CI –0.49 to –0.14; *P*<.001) and heterogeneity of 27.2%. In combined mobile and website group [8,35,39,40,46,47,49,51,53], a heterogeneity of 64.6% was observed, and the pooled effect was –0.77% (95% CI –1.07 to –0.47; *P*<.001). In the group of other intervention types, heterogeneity across studies was 35.5%, with a pooled WMD of –0.23 (95% CI –0.38 to –0.09; *P*=.001; Figure 5).

#### Results of Subgroup Analysis Based on Feedback Provision

**Figure 6:**
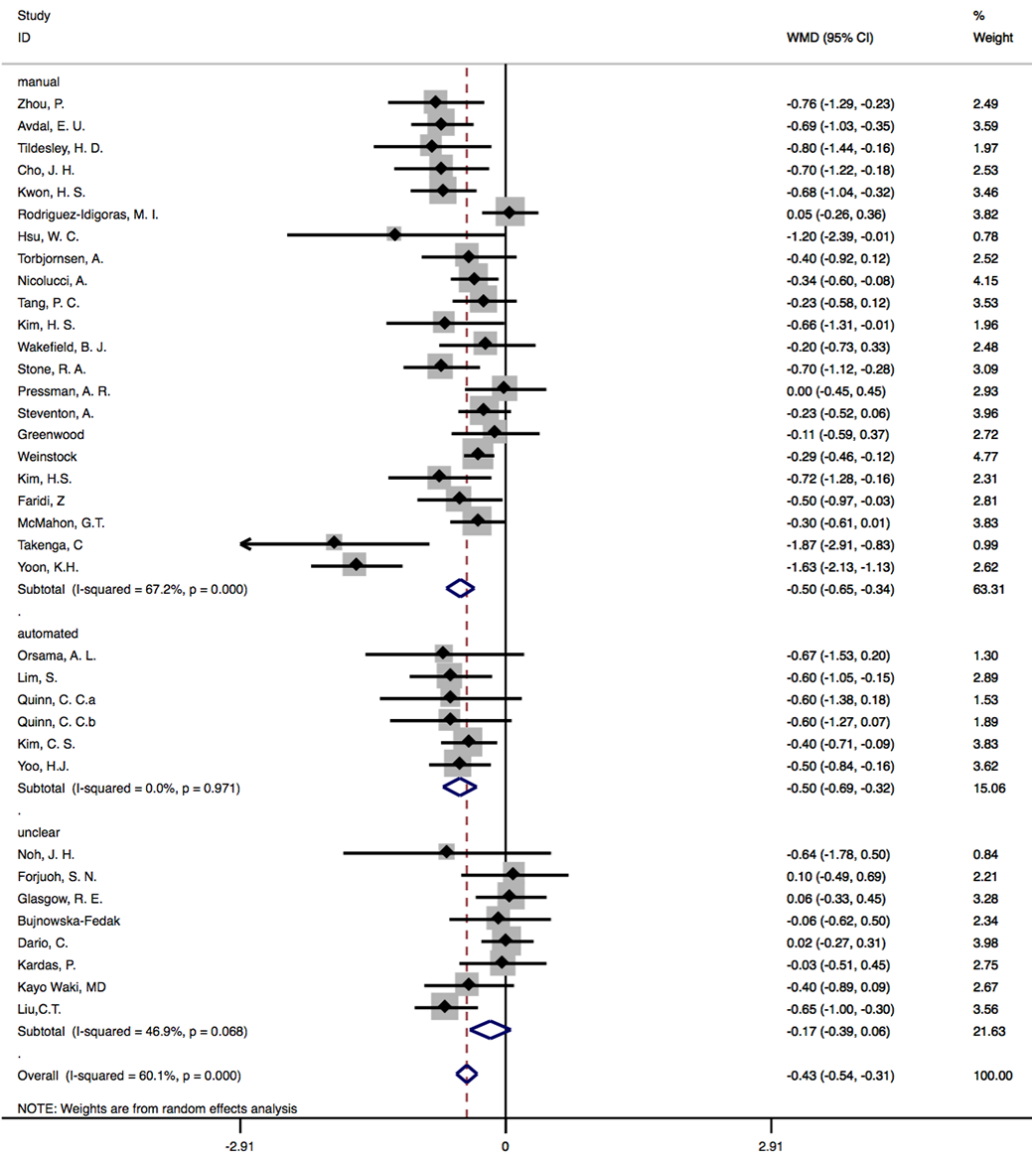
Subgroup analysis based on feedback provision. WMD: weighted mean difference.

We divided included studies into 3 groups (manual: n=22, automated: n=6, and unclear: n=8) to perform subgroup analysis based on feedback provision. A total of 8 studies did not specifically mention ways of providing feedback and thus were classified as unclear. In the group of manual feedback [7,9-11,14,27-32,35,37,40,41,43,44,48-51,53], statistically significant difference in HbA_1c_ between CG and IG was observed, and heterogeneity across studies was 67.2% (WMD –0.50%, 95% CI –0.65 to –0.34; *P*<.001). In studies that provided automated feedbacks [25,36,39,46,52], heterogeneity was reduced to 0.0%, and difference between groups was –0.50% (95% CI –0.69 to –0.32; *P*<.001). In unclear group [8,12,16,26,38,42,45,47], difference in HbA_1c_ outcomes was not significant as *P*=.14, which is greater than .05 (WMD –0.17%, 95% CI –0.39 to –0.06), and heterogeneity across studies was 46.9% (Figure 6).

#### Results of Subgroup Analysis Based on Internet-Based Education Provision

**Figure 7:**
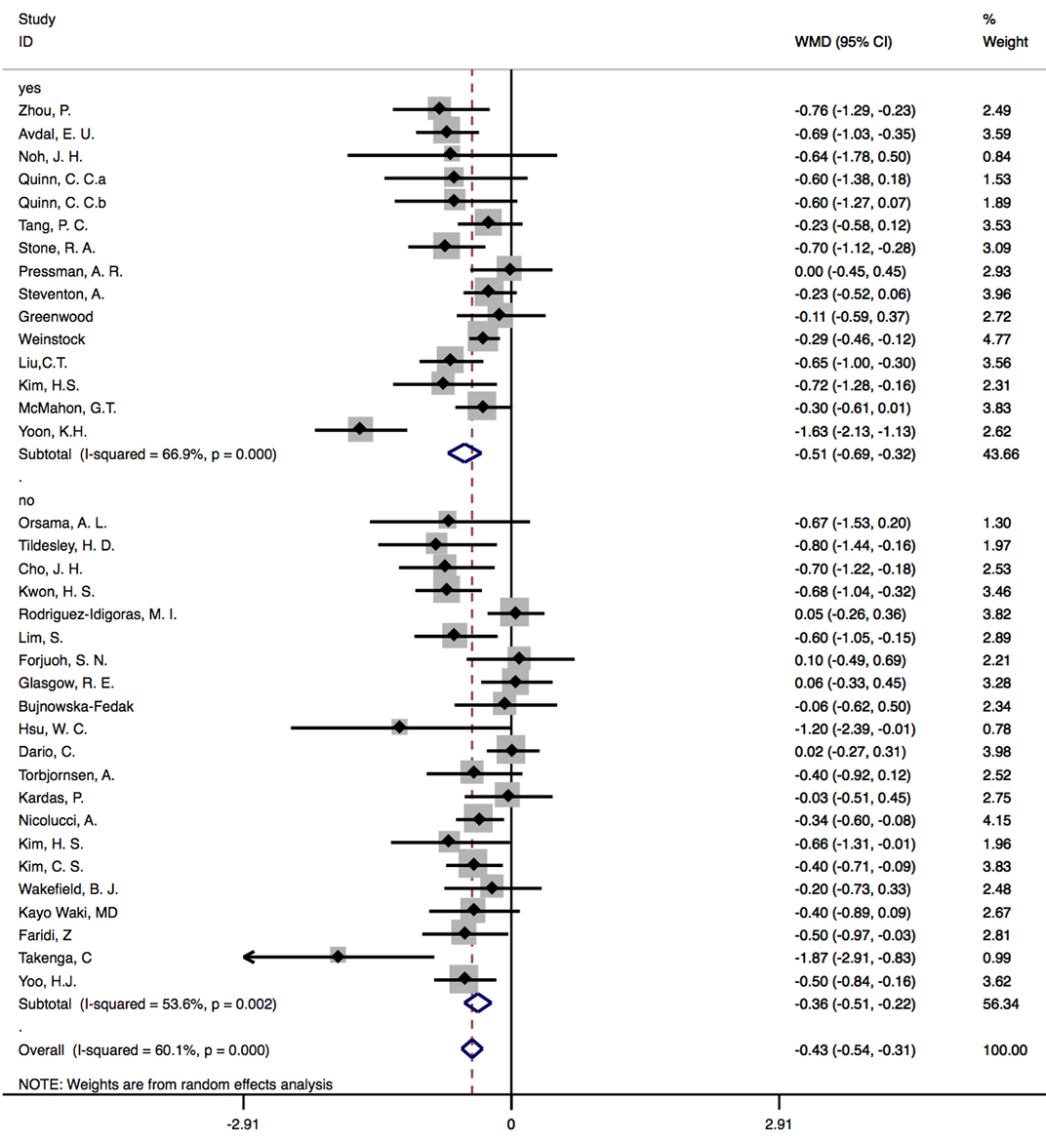
Subgroup analysis based on internet-based education provision. WMD: weighted mean difference.

On the basis of internet-based education provision, 35 studies were divided into 2 subgroups (yes: n=15; no: n=21). In the group that provided internet-based diabetes education [8,10,27,29,32,37,39-41,43,47,49,50,53], there was significant difference between CG and IG, with a pooled WMD of –0.51% (95% CI –0.69 to –0.32; *P*<.001), and heterogeneity across studies was 66.9%. In the group without internet-based education [7,9,11,12,14,16,25,26,28,30,31,35,36,38,42,44-46,48,51,52], heterogeneity decreased to 53.6%, and WMD was –0.36% (95% CI –0.51 to –0.22; *P*<.001; Figure 7).

## Discussion

### Principal Findings

This meta-analysis included 35 RCT studies to evaluate the effect of internet-based interventions on patients with T2DM. WMD of HbA_1c_ value was adopted as the effect indicator of glucose control, and results indicated that the mean difference of HbA_1c_ values between the CG and internet-based interventions was 0.43%, favoring the internet-based interventions. It is reported in research that each 1% decrease of mean HbA_1c_ values will lead to 21% reduction of risk for any endpoint related to diabetes, including death, myocardial infarction, and microvascular complications [54]. Therefore, optimizing the utilization of internet-based interventions in the management of T2DM has great importance.

### Intervention Duration

Duration has a significant influence on the effect of internet-based interventions. Results of this meta-analysis indicate a downward trend of intervention effect with an increasing duration, which is similar to that in other systematic reviews and meta-analyses [19,23]. There are some possible rationales that may serve to explain such differences over time. First, the decline of efficacy may be attributed to patients’ diminishing enthusiasm or motivation with the passage of intervention, especially when most of the studies included in this meta-analysis involve a regular or daily transmission of glucose data. Second, a lack of user-friendliness could also result in attenuated effect. As the mean age of recruited participants exceeds 40 years, new forms of technology may not be familiar and friendly for them. However, this result does not encourage the shortening of intervention periods, but rather it indicates that relative measures should be taken to increase patients’ adherence and maintain their motivation, with the aim to prevent attenuated intervention effect over time. Furthermore, it is promising to develop a form of internet-based intervention that is both effective and friendly to use.

### Types of Internet-Based Interventions

In terms of the types of internet-based interventions, there is not a consensus in previous meta-analyses on which form is the most effective. In this paper, combined website and mobile interventions have the largest WMD of HbA_1c_, followed by Web-based only interventions. Mobile-based only interventions have the lowest mean difference, which indicates that mobile technologies may not be the optimal options in diabetes management. This result coincides with some findings in Toma’s meta-analysis [23]. Toma discovered that the effect of mobile-only interventions in HbA_1c_ outcomes was the poorest and not statistically important (WMD –0.20%, 95% CI –0.43 to 0.03; *P*=.09), whereas combined mobile and websites interventions yielded the best performance (WMD –0.54%, 95% CI –0.72 to –0.37; *P*<.001). Results of websites-only interventions were similar to those in this meta-analysis (WMD –0.51%, 95% CI –0.68 to –0.34). However, in another 2 meta-analyses exploring the effect of mobile-based interventions on diabetes management, the WMD in HbA_1c_ outcomes was –0.40% (95% CI –0.69 to –0.11; *P*=.007) and –0.50% (95% CI –0.7 to –0.3) [22,55]. A possible explanation may be that in most studies included (7 out of 8) in this meta-analysis, a new mobile phone with functions related to diabetes management is provided to participants to replace their own cellular phones, rather than installing a diabetic application in their old ones [14,25,31,42,45,48,52]. Therefore, it is likely that such diabetic phones are not user-friendly and require some time to get familiar with. As a result, patients’ satisfaction toward study and frequency of data transmission may be compromised, which further leads to the compromise of HbA_1c_ performance ultimately. Furthermore, the fact that Pal’s meta-analysis on mobile interventions only included 3 studies and Cui’s meta-analysis also involved some studies that combined the use of mobile phone and websites may also contribute to the contradictory situation [22,55]. However, this result does not imply that we should altogether abandon mobile-based interventions, as it is drawn from the currently available evidence and therefore may lag in time. Due to the high penetration and convenience offered by mobile phones, they have great potentials in diabetes management, and future studies need to explore the effectiveness of apps that are easy to use and directly installed on patients’ own cellular phones.

### Forms of Feedback Provision and Function of Internet-Based Educational Contents

Automated feedbacks generated by algorithms yield similar effect to feedbacks provided manually. As automated algorithms could save manual labor and also provide real-time feedback as soon as patients enter data, it may have great potentials to alleviate the social and economic burden brought by various chronic diseases.

Furthermore, although various clinical guidelines have acknowledged the importance and effectiveness of diabetes education, only 40% of studies included in this meta-analysis embody this feature [4,5]. This result coincides with a previous review where among mobile diabetes apps on the market, only 20% of them had an educational module [3].

### Limitations of This Meta-Analysis

This meta-analysis also has some limitations. First of all, because of limited time, we only searched 3 databases. Although we tried to identify studies from the reference lists of other reviews and include those published in other languages, it is possible that some studies in other databases and gray literature are overlooked. Second, although the studies included in this meta-analysis are all RCTs and the quality of evidence is relatively higher, only a few reported blinding of the outcome assessment process. Therefore, it is possible that performance bias is introduced. Third, in some studies, CGs also use some forms of intervention rather than purely usual care, such as paper-based education, note-keeping of glucose levels, and telephone follow-ups. Furthermore, contents of usual care in different locations may also vary. Therefore, this could also bias the genuine effects of internet-based interventions. Fourth, in some subgroup analyses, results are drawn from a small number of studies and heterogeneity is high. Finally, although the trim-and-fill method did not change the general effect of internet-based interventions, suggesting that the effect was not an artifact of unpublished negative studies, the possibility was not fully ruled out.

### Implications for Future Studies and Clinical Practices

First of all, although blinding of participants and health care providers seems impossible because of the nature of the intervention, only a few studies explicitly reported that the outcome assessment process was blinded. In future studies, more importance should be attached to the blinding of outcome assessment because this is a process that should be achieved regardless of the type interventions. Only in this way, could performance bias be reduced to a minimum and a reliable effect be revealed.

Second, subgroup analysis indicates that automated feedback provision has a similar effect to manual feedback provision; therefore, it is promising to take full advantage of automated algorithms, as it could not only provide real-time feedbacks but also save manual labors. However, the number of studies in this aspect is limited and few have explored its cost-effectiveness and patients’ satisfaction. As a result, future studies need not only examine the efficacy of automated feedback provision but also evaluate the feasibility of promotion on a large scale.

Third, future studies need to integrate diabetes-related functions to existing technologies, such as developing diabetic apps, which could be directly installed into patients’ own mobile phones, rather than developing new types of phones. Only in this way, the true effect of mobile interventions can be revealed.

Finally, results in this meta-analysis also shed some light on clinical practices. If health care practitioners would like to use the internet as a way to help patients manage their glycemic levels, a combined use of websites and mobile technologies may achieve a better outcome than a standalone method. Furthermore, if there are currently available mobile apps on the market, practitioners may as well recommend patients to download such apps into their own mobile phones, rather than developing a new one for them. Moreover, as internet-based health education for diabetes management is proven to be effective, if due to various reasons health care providers could not provide a systematic and comprehensive intervention for patients, they could recommend some reliable and informative websites or information resources with diabetes-related contents that could be easily understood by patients.

### Conclusions

In conclusion, the WMD of endpoint HbA_1c_ between internet-based interventions and usual care was -0.426%. Optimal outcomes appear in intervention duration of ≤3 months. In terms of the types of internet-based interventions, according to currently available evidence, combined interventions of websites and mobile technologies yield better results, and more studies are needed to explore the potentials of mobile technologies. Results also indicate that automated feedback has similar effects to feedbacks provided manually, and interventions with internet-based educational contents have better performance. As a result, interventions integrating the above elements may achieve more satisfactory results and help patients manage their glycemic levels effectively.

## Appendix

Multimedia Appendix 1 Details of search syntax.

Multimedia Appendix 2 Characteristics of eligible studies included in meta-analysis.

Multimedia Appendix 3 Characteristics of intervention group (IG) and control group (CG).

Multimedia Appendix 4 Publication bias.

Multimedia Appendix 5 Risk of bias.

Multimedia Appendix 6 Sensitivity analysis.

Multimedia Appendix 7 HbA_1c_ value and related data of intervention group (IG) and control group (CG) at baseline and different intervention periods.

## Abbreviations

BMI: body mass index
CG: control group
HbA_1c_: glycated hemoglobin
IG: intervention group
RCT: randomized controlled trial
T2DM: type 2 diabetes mellitus
WMD: weighted mean difference
