# Supplementary material for: Effectiveness of Internet-Based Interventions on Glycemic Control in Patients With Type 2 Diabetes: Meta-Analysis of Randomized Controlled Trials
Source: J Med Internet Res. 2018 May 7;20(5):e172. doi: 10.2196/jmir.9133 (PMC5962831; doi:10.2196/jmir.9133)
Supplement: Multimedia Appendix 1 [file jmir_v20i5e172_app1.pdf]

Database	Search Syntax
Pubmed	((diabetes AND Type 2) OR T2DM) AND (social media OR e-learning OR new media OR m-health OR mHealth OR internet-based OR web-based OR twitter OR eHealth OR e-health OR Facebook OR computer-delivered OR social web OR social software OR online case-based learning OR mobile learning OR digital game-based learning OR serious games OR wearable devices OR mobile APP OR mobile application OR smart phone-based) AND (RCT OR Randomized Controlled Trial)
Sciencedirect	(tak(social media) or tak(e-learning) or tak(new media) or tak(m-health) or tak(mHealth) or tak(internet-based) or tak(web-based) or tak(twitter) or tak(eHealth) or tak(e-health) or tak(Facebook) or tak(computer-delivered) or tak(social web) or tak(social software) or tak(online case-based learning) or tak(mobile learning) or tak(digital game-based learning) or tak(serious games) or tak(wearable devices) or tak(mobile APP) or tak(mobile application) or tak(smart phone-based)) AND (tak(RCT) or tak(Randomized Controlled Trial)) AND ((tak(diabetes) AND tak(type 2)) or tak(T2DM))
Web of Science	(TS=((social media OR e-learning OR new media OR m-health OR mHealth OR internet-based OR web-based OR twitter OR e-health OR eHealth OR Facebook OR computer-delivered OR social web OR social software OR online case-based learning OR mobile learning OR digital game-based learning OR serious games OR wearable devices OR mobile APP OR mobile application OR smart phone-based) AND ((diabetes AND Type 2) OR T2DM) AND (RCT OR Randomized Controlled Trial)))

Multimedia appendix 1. Details of search syntax
