# Supplementary material for: Effectiveness of Internet-Based Interventions on Glycemic Control in Patients With Type 2 Diabetes: Meta-Analysis of Randomized Controlled Trials
Source: J Med Internet Res. 2018 May 7;20(5):e172. doi: 10.2196/jmir.9133 (PMC5962831; doi:10.2196/jmir.9133)
Supplement: Multimedia Appendix 2 [file jmir_v20i5e172_app2.pdf]

Multimedia appendix 2: Characteristics of eligible studies included in meta-analysis

No., author, year	Number of participants	Participant characteristics	Study design, duration, location
1. Zhou, P., 2014 [1]	In total: 114; IG[1]: 57; CG[2]: 57<br>CR[3]: 94.7%;<br>IG:53 Attrition: 7.0%;<br>CG: 55 Attrition: 3.5%	Patients: diagnosed as type 2 Diabetes according to WHO criteria in 1999 and aged 18 to 75 years old who can manage glycemic meter at home and voluntarily participate in the trial<br>Mean age:-<br>Male (%):-<br>BMI (kg/m2): CG: 23.64 (3.01); IG: 24.72 (3.38)<br>Other features: patients were referred to the First Affiliated Hospital of Jinan University from July 2012 to February 2013	RCT, 3 months, China
2. Orsama, A. L., 2013 [2]	In total: 56; IG: 27; CG: 29<br>CR: 94.6%;<br>IG: 24 Attrition: 11.1%;<br>CG: 24 Attrition: 17.3%	Patients: known diagnosis of type 2 diabetes, elevated HbA1c (6.5-11%) or use of oral diabetes medication, and age range of 30-70 years<br>Mean age: CG: 61.5 (9.1); IG: 62.3 (6.5)<br>Male (%): 54% (CG&IG)<br>BMI (kg/m2): CG: 33.5 (8.0); IG: 30.7 (4.5)<br>Other features: -	RCT, 10 months, Finland

1 IG: intervention group
2 CG: control group
3 CR: completion rate

No., author, year	Number of participants	Participant characteristics	Study design, duration, location
3. Avdal, E. U., 2011 [3]	In total: 122; IG: 61; CG: 61<br>CR: 100%;<br>IG: 61 Attrition: 0%;<br>CG: 61 Attrition: 0%	Patients: older than 18 years old, had the diagnosis of T2DM at least for 6 months, underwent insulin treatment, had an A1c level greater than 7%, without advanced level retinopathy and neuropathy, had to be able to use a computer and the Internet, and volunteered to participate in this study.<br>Mean age: CG: 52.45 (7.01); IG: 50.59 (7.5)<br>Male (%): CG: 49.18%; IG: 49.18%<br>BMI (kg/m2):-<br>Other features: patients were registered in the Endocrine Polyclinic, Dokuz Eylul University and completed basic diabetes education provided by diabetes nurses.	RCT, 6 months, Turkey
4. Noh, J. H., 2010 [4]	In total: 44; IG: 24; CG: 20<br>6 months after the intervention: CR: 91%;<br>IG: 20 Attrition: 16.7%;<br>CG: 20 Attrition: 0%	Patients: 18– 80 years old with type 2 diabetes either drug naive or who had received prior drug therapy and had a glycated hemoglobin (A1C) level between 7% and 10% with stable glycemic control.<br>Mean age: CG: 42.3 (7.6); IG: 42.5 (10.6)<br>Male (%): CG: 75%; IG: 80%<br>BMI (kg/m2): CG: 24.7 (2.8); IG: 25.7 (3.1)<br>Other features: Persons participating in this study had Internet assess in their homes, their own cellular phone, and the ability to access the Internet and mobile web site.	RCT, 7 months, Republic of Korea
5. Tildesley, H. D., 2010 [5]	In total: 50; IG: 25; CG: 25<br>CR: 94%;<br>IG: 24 Attrition: 4%;<br>CG: 23 Attrition: 8%	Patients: treated with insulin alone or in combination with oral anti-hyperglycemic medications, recent A1C >7.0%, had Internet access and prior training in self-monitoring of blood glucose (SMBG).<br>Mean age: CG: 62 (7.2); IG: 57 (10)<br>Male (%): CG: 65.2%; IG: 58.3%	RCT, 6 months, Canada

No., author, year	Number of participants	Participant characteristics	Study design, duration, location
		BMI (kg/m2): CG: 33.1 (6.0) IG: 33.7 (6.4)<br>Other features: -	
6. Cho, J. H., 2006 [6]	In total: 80; IG: 40; CG: 40<br>CR: 88.9%;<br>IG: 35 Attrition: 12.5%;<br>CG: 36 Attrition: 10%	Patients: type 2 diabetes patients ≥30 years of age who had been followed up for 6 months in Kangnam St. Mary's Hospital Diabetes Center.<br>Mean age: CG: 54.6 (8.6); IG: 51.3 (9.1)<br>Male (%): CG: 57.5%; IG: 65%<br>BMI (kg/m2): CG: 23.8 (2.8); IG: 22.8 (2.6)<br>Other features: those who did not have Internet access in their homes or offices, did not know how to use the Internet, did not wish to participate in the study, had participated in similar studies or received diabetes management education from any Web site other than our own were excluded.	RCT, 30 months, Republic of Korea
7. Kwon, H. S., 2004 [7]	In total: 110; IG: 55; CG: 55<br>CR: 91.8%;<br>IG: 51 Attrition: 7.27%;<br>CG: 50 Attrition: 10%	Patients: Men and women diagnosed with type 2 diabetes for 1 year, ≥30 years of age, had Internet access.<br>Mean age: CG: 54.7 (9.4); IG: 53.5 (8.8)<br>Male (%): CG: 58.2%; IG: 63.6%<br>BMI (kg/m2): CG: 23.9 (3.1); IG: 24.4 (3.4)<br>Other features: Patients were recruited from the outpatient clinic of Kangnam St. Mary's Hospital Diabetes Center between May and August 200l.	RCT, 3 months, Republic of Korea

No., author, year	Number of participants	Participant characteristics	Study design, duration, location
8. Rodriguez-Idigoras, M. I., 2009 [8]	In total: 328; IG: 161; CG: 167<br>at 6 months: CR: 94.2%;<br>IG: 149 Attrition: 7.5%;<br>CG: 160 Attrition: 4.2%<br>at 12 months: CR: 90.5%;<br>IG: 146 Attrition: 9%;<br>CG: 151 Attrition: 10%	Patients: >30 years of age, diagnosed with type 2 diabetes and on self-monitoring for at least 6 months before the beginning of the study.<br>Mean age: CG: 64.52 (62.96, 66.09); IG: 63.32 (61.60, 65.04) (95% CI, p=0.307)<br>Male (%): CG: 49.10%; IG: 54.04%<br>BMI (kg/m2):-<br>Other features: patients were under the care of 35 family physicians in the province of Malaga, Spain, who agreed to participated in the study. Physicians agreed to participate first, patients were selected afterwards.	cluster RCT, 12 months, Spain
9. Lim, S., 2016 [9]	In total: 100; IG: 50; CG: 50<br>CR: 85%; IG: 43 Attrition: 14%; CG: 42 Attrition: 16%	Patients: Patients with type 2 diabetes, aged 60 years or older with HbA1c level of 7.0–10.5 %, able to use text messages or to access the internet.<br>Mean age: CG: 65.8 (4.7); IG: 64.3 (5.2)<br>Male (%): CG: 70%; IG: 80%<br>BMI (kg/m2): CG: 25.4 (3.3); IG: 25.9 (3.6)<br>Other features: Patients were recruited from the outpatient clinic of the Seoul National University Bundang Hospital from December, 2013 to January, 2014.	RCT, 6 months, Republic of Korea
10. Forjuoh, S. N., 2014 [10]	In total: 176; IG: 81; CG: 95<br>at 6 months: CR: 85.2%;<br>IG: 64 Attrition: 21%;<br>CG: 86 Attrition: 9.5%<br>at 12 months: CR: 68.2%;<br>IG: 47 Attrition: 42%;	Patients: had a diagnosis of T2DM; were ≥18 years; had a lab assessed HbA1c value ≥7.5% within the last six months; and were able to communicate in English.<br>Mean age: CG: 58.5 (11.9); IG: 57.7 (10.8)<br>Male (%): CG: 44.2%; IG: 42%<br>BMI (kg/m2): CG: 33.9 (7.7); IG: 35.3 (7.3)<br>Other features: Participants were recruited from seven participating clinics in Central Texas. These clinics had high percentage of African American and Hispanic patients.	RCT, 12 months, the United States

No., author, year	Number of participants	Participant characteristics	Study design, duration, location
	CG: 73 Attrition: 23.2%	African American and Hispanic (%): CG: 33.7%; IG: 37%	
11. Glasgow, R. E., 2010 [11]	In total: 301; IG: 169; CG: 132<br>at 4 months: CR: 81.4%;<br>IG: 130 Attrition: 23.1%;<br>CG: 115 Attrition: 12.9%<br>at 12 months: CR: 77.7%;<br>IG: 120 Attrition: 29%;<br>CG: 114 Attrition: 13.6%	Patients: 25–75 years of age, diagnosis of type 2 diabetes, body mass index (BMI) of 25 kg/m2 or greater, and at least one other risk factor for heart disease, access to a telephone and at least biweekly access to the Internet, ability to read and write in English or Spanish, and to perform mild to moderate physical activity.<br>Mean age: CG: 58.7 (9.1); IG: 58.7 (9.3)<br>Male (%): CG: 48.5%; IG: 55.4%<br>BMI (kg/m2): CG: 34.77 (6.55); IG: 34.47 (6.28)<br>Other features: The study was conducted in five primary care clinics within Kaiser Permanente Colorado. Latino ethnicity: 21%; African American: 14%	RCT, 12 months, the United States
12. Quinn, C. C., 2011 [12]	In total: 175; IG1: 33; IG2: 80; CG: 62<br>CR: 80%;<br>IG1: 22 Attrition: 33.3%;<br>IG2: 62 Attrition 22.5%;<br>CG: 56 Attrition: 19.4%	Patients: Physician diagnosis of type 2 diabetes for 6 months; HbA1c≥7.5% within 3 months; Age 18–64 years.<br>Mean age: CG: 53.2 (8.4); IG1: 53.7 (8.2); IG2: 52 (8.0)<br>Male (%): CG: 50%; IG1: 45.5%; IG2: 50%<br>BMI (kg/m2): CG: 34.3 (6.3); IG1: 35.5 (10.3); IG2: 35.8 (7.1)<br>Other features: Only patients covered by commercial insurer were eligible. Primary care practices were selected first and randomly assigned to one group.	RCT, 12 months, the United States
13. Bujnowska-Fedak, 2011 [13]	In total: 100; IG: 50; CG: 50<br>CR: 95%;<br>IG: 47 Attrition: 6%;	Patients: 18-75 years old with T2DM. IG: had a PC at home with Internet access.<br>Mean age: CG: 57.5 (27.4); IG: 53.1 (25.2)<br>Male (%): CG: 52.1%; IG: 55.3%<br>BMI (kg/m2): CG: 26.2 (6.6); IG: 25.4 (7.2)	RCT, 6 months, Poland

No., author, year	Number of participants	Participant characteristics	Study design, duration, location
	CG: 48 Attrition: 4%	Other features: all were regular patients of general practitioners in the Lower Silesia Region in Poland between 2007 and 2009. Patients were equally and purposefully divided between insulin and noninsulin requiring.	
14. Hsu, W. C., 2016 [14]	In total: 40; IG: 20; CG: 20<br>CR: 87.5%;<br>IG: 19 Attrition: 5%;<br>CG: 16 Attrition: 20%	Patients: ≥18 years of age with HbA1c levels of 9–14%, who were being started on basal insulin therapy by their treating HCPs and had internet connectivity.<br>Mean age: CG: 53.8; IG: 53.3<br>Male (%): -<br>BMI (kg/m2): CG: 31.7; IG: 30.8<br>Other features: staggered recruitment took place in a tertiary diabetes center in Massachusetts.	RCT, 12±2 weeks, the United States
15. Dario, C., 2016 [15]	In total: 299; IG: 208; CG: 91<br>CR: 81.3%;<br>IG: 166 Attrition: 20.2%;<br>CG: 77 Attrition: 15.4%	Patients: with a diagnosis of T2DM already confirmed and HbA1c > 7.0%<br>Mean age: CG: 73.04 (5.28); IG: 73.05 (5.79)<br>Male (%): CG: 53%; IG: 57%<br>BMI (kg/m2):-<br>Other features: the study was part of the RENEWNING HEALTH project in Europe.	RCT, 12 months, Italy
16. Torbjornsen, A., 2014 [16]	In total: 101; IG: 51; CG: 50<br>4 months: CR: 85.1%;<br>IG: 42 Attrition: 17.6%;<br>CG: 44 Attrition: 12%<br>12 months: CR: 79.2%;<br>IG: 39 Attrition: 23.5%;	Patients: age ≥18 years, diagnosed with T2DM a minimum of 3 months before inclusion, HbA1c ≥7.1%, able to use the FTA system, and capable of understanding and completing the questionnaires.<br>Mean age: CG: 55.9 (12.2); IG: 58.6 (11.8)<br>Male (%): CG: 60%; IG: 67%<br>BMI (kg/m2): CG: 32.0 (6.0); IG: 32.4 (6.5)<br>Other features: the study was part of the RENEWNING HEALTH project in Europe.	RCT, 12 months, Norway

No., author, year	Number of participants	Participant characteristics	Study design, duration, location
	CG: 41 Attrition: 18%		
17. Kardas, P., 2016 [17]	In total: 62; IG: 32; CG: 30<br>CR: 96.8%;<br>IG: 30 Attrition: 6.25%;<br>CG: 30 Attrition: 0%	Patients: age 18–65 years, T2DM diagnosed ≥6 months prior to the study, currently in the maintenance phase of treatment, self-committed ability to use the cell phone and the sensors.<br>Mean age: CG: 59.0 (8.09); IG: 59.9 (5.31)<br>Male (%): CG: 63.3%; IG: 56.7%<br>BMI (kg/m2): CG: 30.3 (3.35); IG: 31.6 (5.27)<br>Other features: -	RCT, 6 weeks, Poland
18. Nicolucci, A., 2015 [18]	In total: 302; IG: 153; CG: 149<br>CR: 82.5%;<br>IG: 114 Attrition: 25.5%;<br>CG: 135 Attrition: 9.4%	Patients: with T2DM, >45 years old, HbA1c between 7.5%-10%, BP>130/80mm Hg regardless of the presence of medications<br>Mean age: CG: 57.8 (8.9); IG: 59.1 (10.3)<br>Male (%): CG: 61.7; IG: 61.4<br>BMI (kg/m2): CG: 29.0 (5.0); IG: 28.7 (4.6)<br>Other features: patients were in treatment with sulfonylureas alone or in association with other oral hypoglycemic agents, or treated with basal insulin alone or in association with oral hypo- glycemic agents	RCT, 12 months, Italy
19. Tang, P. C., 2013 [19]	In total: 415; IG: 202; CG: 213<br>at 6 months: CR: 90.1%;<br>IG: 185 Attrition: 8.4%;<br>CG: 189 Attrition: 11.3%<br>at 12 months: CR: 91.3%;	Patients: ≥18 years old, T2DM diagnosis≥12 months, A1C≥7.5%, patients seen within the past 12 months at Palo Alto Medical Foundation (PAMF), spoke English, insured, had regular internet access with email capabilities<br>Mean age: CG: 53.5 (10.2); IG: 54.0 (10.7)<br>Male (%): CG: 61%; IG: 58.9%<br>BMI (kg/m2): -	RCT, 12 months, the United States

No., author, year	Number of participants	Participant characteristics	Study design, duration, location
	IG: 186 Attrition: 7.9%;<br>CG:193 Attrition: 9.4%	Other features: patients were screened through PAMF's EHRs	
20. Kim, H. S., 2007 [20]	In total: 60; IG: 30; CG: 30<br>CR: 85%;<br>IG: 25 Attrition: 16.7%;<br>CG: 26 Attrition: 13.3%	Patients: with T2DM, participants should be able to perform blood glucose self-testing and self-injection of medication, access websites and have their own cellular phone.<br>Mean age: CG: 47.5 (9.1); IG: 46.8 (8.8)<br>Male (%): CG: 42.3%; IG: 44%<br>BMI (kg/m2): CG: 23.4 (2.5); IG: 24.5 (2.9)<br>Other features: Patients were recruited from the endocrinology outpatient department of a tertiary care hospital in South Korea.	RCT, 6 months, Republic of Korea
21. Kim, C. S., 2010 [21]	In total: 100; IG: 50; CG: 50<br>CR: 92%;<br>IG: 47 Attrition: 6%;<br>CG: 45 Attrition: 20%	Patients: age ≥18 years, taking anti-diabetes treatment for >6 months, requiring a long-acting insulin therapy, HbA1C: 7%-12%, BMI<35 kg/m2<br>Mean age: CG: 49.0 (10.7); IG: 47.8 (9.6)<br>Male (%): CG: 48.9%; IG: 51.1%<br>BMI (kg/m2): CG: 24.4 (3.5); IG: 23.6 (2.5)<br>Other features: patients were recruited from the outpatient clinic of Hallym University Sacred Heart Hospital.	RCT, 3 months, Republic of Korea
22. Wakefield, B. J., 2014 [22]	In total: 108; IG: 53; CG: 55<br>CR: 76.7%;<br>IG: 40 Attrition: 24.5%;<br>CG: 43 Attrition: 21.8%	Patients: T2DM diagnosis ≥1 year, were out of desired range on either A1c (≥8%) or SBP (>130 mm Hg), age ≥18 years old, taking either oral diabetes medication or injectable insulin, currently using and/or owning a BG meter compatible with study equipment, having an in-home analog phone line or computer with Internet connection, receiving primary care at FM or GIM clinics and anticipating receiving primary care	RCT, 3 months, Republic of Korea

No., author, year	Number of participants	Participant characteristics	Study design, duration, location
		for the next 12 months from that clinic<br>Mean age: CG: 62.5 (10.9); IG: 57.7 (10.8)<br>Male (%): CG: 41%; IG: 47%<br>BMI (kg/m2): -<br>Other features: most people were enrolled based on out-of-control BP	
23. Stone, R. A., 2010 [23]	In total: 150; IG: 73; CG: 77<br>CR: 91.3%;<br>IG: 64 Attrition: 12.3%;<br>CG: 73 Attrition: 5.2%	Patients: had at least one outpatient visit in a primary care clinic between 1 June 2004 and 31 December 2005, were aged<80 years, received pharmacological treatment for diabetes for 12 months, had no referrals to the VAPHS Diabetes Clinic in the preceding 18 months, and point- of-care capillary A1C ≥7.5% at enrollment.<br>Mean age: -<br>Male (%): CG: 97.3%; IG: 100%<br>BMI (kg/m2): -<br>Other features: participants were veterans, vast majority were male and non-Hispanic white	RCT, 6 months, the United States
24. Pressman, A. R., 2014 [24]	In total: 225; IG: 118; CG: 107<br>CR: 88%;<br>IG: 107 Attrition: 9%;<br>CG: 91 Attrition: 15%	Patients: 18–75 years old with T2DM diagnosed for at least 1 month who were referred to the diabetes care management program and had HbA1c values between 7.5% and 10.5%.<br>Mean age: CG: 56.4 (8.7); IG: 54.8 (9.8)<br>Male (%): CG: 60%; IG: 63%<br>BMI (kg/m2): CG: 35.5 (6.0); IG: 34.8 (6.7)<br>Other features: -	RCT, 6 months, the United States
25. Steventon, A.,	In total: 513; IG: 300; CG:	Patients: ≥18 years old, had a diagnosis of diabetes, had a telephone line and electricity	RCT, 12 months,

No., author, year	Number of participants	Participant characteristics	Study design, duration, location
2014 [25]	213<br>CR: -%;<br>IG: - Attrition: -%;<br>CG:- Attrition: -%	Mean age: CG: 66.2 (11.9); IG: 63.9 (13.0)<br>Male (%): CG: 64.3%; IG: 53.3%<br>BMI (kg/m2): CG: 30.3 (5.9); IG: 31.8 (6.6)<br>Other features: The current study was conducted on a subset of WSD patients who had diabetes as their "index condition". WSD trial was conducted in many primary practices and interventions in different sites might vary.	England
26. Waki, K., MD, 2014 [26]	In total: 54; IG: 27; CG: 27<br>CR: 90.7%;<br>IG: 24 Attrition: 11.1%;<br>CG: 25 Attrition: 7.4%	Patients: without severe complications, able to exercise, T2DM history more than 5 years, no HbA1c limits<br>Mean age: CG: 57.4 (9.4); IG: 57.1 (10.2)<br>Male (%): CG: 77.8%; IG: 74.1%<br>BMI (kg/m2): -<br>Other features:	RCT, 3 months, Japan
27. Greenwood, D. A., 2015 [27]	In total: 90; IG: 45; CG: 45<br>CR: 90%;<br>IG: 40 Attrition: 12.5%;<br>CG: 41 Attrition: 8.9%	Patients: T2DM patients treated with oral medications, noninsulin injectable medications or lifestyle alone; 30-70 years old; HbA1c between 7.5%-10.9% in previous 6 months; Internet or 3G connection with email access; Landline or cellular phone; English-speaking.<br>Mean age: CG: 57.5 (10.6); IG: 53.9 (10.4)<br>Male (%): CG: 79%; IG: 75%<br>BMI (kg/m2): CG: 34.1 (6.6); IG: 34.1 (6.8)<br>Other features: Patients were participants in a diabetes management program in California for previous 12 months; non-White: 36%; participants were highly educated and employed.	RCT, 6 months, the United States

No., author, year	Number of participants	Participant characteristics	Study design, duration, location
28. Weinstock, R. S., 2011 [28]	In total: 1665; IG: 844; CG: 821<br>CR: 47.6%;<br>IG: 330 Attrition: 60.9%;<br>CG: 463 Attrition: 43.6%	Patients: medicare beneficiaries lived in medically underserved area, ≥55 years old, on treatment with diet, an oral hypoglycemic agent or insulin.<br>Mean age: CG: 79.9 (6.8); IG: 70.8 (6.5)<br>Male (%): CG: 37.9%; IG: 36.5%<br>BMI (kg/m2): -<br>Other features: 50% white, 15% black and 35% Hispanic	RCT, 5 years, the United States
29. Liu, C.T., 2005	In total: 274; IG: 140; CG: 134<br>CR: 100%;<br>IG: 140 Attrition: 0%;<br>CG: 134 Attrition: 0%	Patients: type 2 diabetic patients were recruited from hospital<br>Mean age: CG: 61.2 (12); IG: 66 (8.5)<br>Male (%): CG: 46%; IG: 57%<br>BMI (kg/m2): -<br>Other features: -	RCT, 8 months, China
30. Kim, H.S., 2008	In total: 60; IG: 30; CG: 30<br>CR: 56.7%;<br>IG: 18 Attrition: 40%;<br>CG: 16 Attrition: 46.7%	Patients: Participants were recruited from the endocrinology outpatient department of a tertiary care hospital located in an urban city of South Korea.<br>Mean age: CG: 47.5 (9.1); IG: 46.8 (8.8)<br>Male (%): CG: 43.8%; IG: 50%<br>BMI (kg/m2): CG: 25 ± 1.7; IG: 25.6 ± 2.4<br>Other features: -	RCT, 6 months, Republic of Korea
31. Faridi, Z., 2008	In total: 30; IG: 15; CG: 15<br>CR: 100%;<br>IG: 15 Attrition: 0%;<br>CG: 15 Attrition: 0%	Patients: age ≥18 years; type 2 diabetes diagnosed by a health professional at least 1 year prior and confirmed by other clinical laboratory data; controlled by either diet or oral medications for at least 3 months<br>Mean age: CG: 56.7 (10.6); IG: 55.3 (8.7)<br>Male (%): CG: 33.3%; IG: 40%	RCT, 3 months, United States

No., author, year	Number of participants	Participant characteristics	Study design, duration, location
		BMI (kg/m2): CG: 36.9 (12.5); IG: 34.3 (7.4)<br>Other features: -	
32. McMahon, G.T., 2005	In total: 104; IG: 52; CG: 52<br>CR: 100%;<br>IG: 52 Attrition: 0%;<br>CG: 52 Attrition: 0%	Patients: HbA1c ≥9.0%, age >18 years, ability to understand written and spoken English, and willingness to use a notebook computer, glucose and blood pressure monitoring devices<br>Mean age: CG: 63 ± 7; IG: 64 ± 7<br>Male (%): CG: 100%; IG: 99%<br>BMI (kg/m2): CG: 34.1 ± 7.0; IG: 32.3 ± 5.6<br>Other features: -	RCT, 12 months, United States
33. Takenga, C., 2014	In total: 40; IG: 20; CG: 20<br>CR: 77.5%;<br>IG: 17 Attrition: 15%;<br>CG: 14 Attrition: 30%	Patients: Patients diagnosed with type 2 diabetes and aged between 35 and 75 years were recruited randomly.<br>Mean age: CG: 53.4 (9.6); IG: 53.3 (10.7)<br>Male (%): -<br>BMI (kg/m2): -<br>Other features: -	RCT, 2 months, Congo
34. Yoo, H.J., 2009	In total: 123; IG: 62; CG: 61<br>CR: 90.2%;<br>IG: 57 Attrition: 8.1%;<br>CG: 54 Attrition: 11.5%	Patients: between 30 and 70 years of age; a diagnosis of both Type 2 diabetes and hypertension at least 1 year previously by a physician; HbA1c 6.5–10.0%; blood pressure > 130 ⁄ 80 mmHg; BMI ≥ 23.0 kg ⁄ m2<br>Mean age: CG: 59.4 (8.4); IG: 57.0 (9.1)<br>Male (%): CG: 64.8%; IG: 52.6%<br>BMI (kg/m2): CG: 25.5 ± 3.3; IG: 25.6 ± 3.5<br>Other features: -	RCT, 3 months, Republic of Korea

No., author, year	Number of participants	Participant characteristics	Study design, duration, location
35. Yoon, K.H., 2008	In total: 60; IG: 30; CG: 30<br>CR: 85%;<br>IG: 25 Attrition: 16.7%;<br>CG: 26 Attrition: 13.3%	Patients: participants should be able to perform blood glucose self-testing and access websites and have their own cellular phone.<br>Mean age: CG: 47.5 (9.1); IG: 46.8 (8.8)<br>Male (%): CG: 42.3%; IG: 44%<br>BMI (kg/m2): CG: 23.4 ± 2.5; IG: 24.5 ± 2.9<br>Other features: -	RCT, 12 months, Republic of Korea

Reference

1. Zhou P, Xu L, Liu X, Huang J, Xu W, Chen W. Web-based telemedicine for management of type 2 diabetes through glucose uploads: a randomized controlled trial. Int J Clin Exp Pathol 2014;7:8848-8854. PMID: 25674254
2. Orsama AL, Lahteenmaki J, Harno K, Kulju M, Wintergerst E, Schachner H, Stenger P, Leppanen J, Kaijanranta H, Salaspuro V, Fisher WA. Active assistance technology reduces glycosylated hemoglobin and weight in individuals with type 2 diabetes: results of a theory-based randomized trial. Diabetes Technol Ther 2013;15:662-669. PMID: 23844570
3. Avdal EU, Kizilci S, Demirel N. The effects of web-based diabetes education on diabetes care results: a randomized control study. Comput Inform Nurs 2011;29:101-106. PMID: 21099675
4. Noh JH, Cho YJ, Nam HW, Kim JH, Kim DJ, Yoo HS, Kwon YW, Woo MH, Cho JW, Hong MH, Yoo JH, Gu MJ, Kim SA, An KE, Jang SM, Kim EK, Yoo HJ. Web-based comprehensive information system for self-management of diabetes mellitus. Diabetes Technol Ther 2010;12:333-337. PMID: 20388042
5. Tildesley HD, Mazanderani AB, Ross SA. Effect of Internet therapeutic intervention on A1C levels in patients with type 2 diabetes treated with insulin. Diabetes Care 2010;33:1738-1740. PMID: 20668152
6. Cho JH, Chang SA, Kwon HS, Choi YH, Ko SH, Moon SD, Yoo SJ, Song KH, Son HS, Kim HS, Lee WC, Cha BY, Son HY, Yoon KH. Long-term effect

of the Internet-based glucose monitoring system on HbA1c reduction and glucose stability: a 30-month follow-up study for diabetes management with a ubiquitous medical care system. Diabetes Care 2006;29:2625-2631. PMID: 17130195

7. Kwon HS, Cho JH, Kim HS, Song BR, Ko SH, Lee JM, Kim SR, Chang SA, Kim HS, Cha BY, Lee KW, Son HY, Lee JH, Lee WC, Yoon KH. Establishment of blood glucose monitoring system using the internet. Diabetes Care 2004;27:478-483. PMID: 14747232

8. Rodriguez-Idigoras MI, Sepulveda-Munoz J, Sanchez-Garrido-Escudero R, Martinez-Gonzalez JL, Escolar-Castello JL, Paniagua-Gomez IM, Bernal-Lopez R, Fuentes-Simon MV, Garofano-Serrano D. Telemedicine influence on the follow-up of type 2 diabetes patients. Diabetes Technol Ther 2009;11:431-437. PMID: 19580356

9. Lim S, Kang SM, Kim KM, Moon JH, Choi SH, Hwang H, Jung HS, Park KS, Ryu JO, Jang HC. Multifactorial intervention in diabetes care using real-time monitoring and tailored feedback in type 2 diabetes. Acta Diabetol 2016;53:189-198. PMID: 25936739

10. Forjuoh SN, Bolin JN, Huber JC, Jr., Vuong AM, Adepoju OE, Helduser JW, Begaye DS, Robertson A, Moudouni DM, Bonner TJ, McLeroy KR, Ory MG. Behavioral and technological interventions targeting glycemic control in a racially/ethnically diverse population: a randomized controlled trial. BMC Public Health 2014;14:71. PMID: 24450992

11. Glasgow RE, Kurz D, King D, Dickman JM, Faber AJ, Halterman E, Wooley T, Toobert DJ, Strycker LA, Estabrooks PA, Osuna D, Ritzwoller D. Outcomes of minimal and moderate support versions of an internet-based diabetes self-management support program. J Gen Intern Med 2010;25:1315-1322. PMID: 20714820

12. Quinn CC, Shardell MD, Terrin ML, Barr EA, Ballew SH, Gruber-Baldini AL. Cluster-randomized trial of a mobile phone personalized behavioral intervention for blood glucose control. Diabetes Care 2011;34:1934-1942. PMID: 21788632

13. Bujnowska-Fedak MM, Puchala E, Steciwko A. The impact of telehome care on health status and quality of life among patients with diabetes in a primary care setting in Poland. Telemed J E Health 2011;17:153-163. PMID: 21375410

14. Hsu WC, Lau KH, Huang R, Ghiloni S, Le H, Gilroy S, Abrahamson M, Moore J. Utilization of a Cloud-Based Diabetes Management Program for Insulin Initiation and Titration Enables Collaborative Decision Making Between Healthcare Providers and Patients. Diabetes Technol Ther 2016;18:59-67. PMID: 26645932

15. Dario C, Toffanin R, Calcaterra F, Saccavini C, Stafylas P, Mancin S, Vio E. Telemonitoring of Type 2 Diabetes Mellitus in Italy. Telemedicine journal and e-health : the official journal of the American Telemedicine Association 2016. PMID: 27379995

16. Torbjornsen A, Jenum AK, Smastuen MC, Arsand E, Holmen H, Wahl AK, Ribu L. A Low-Intensity Mobile Health Intervention With and Without

Health Counseling for Persons With Type 2 Diabetes, Part 1: Baseline and Short-Term Results From a Randomized Controlled Trial in the Norwegian Part of RENEWING HEALTH. JMIR Mhealth Uhealth 2014;2:e52. PMID: 25499592
17. Kardas P, Lewandowski K, Bromuri S. Type 2 Diabetes Patients Benefit from the COMODITY12 mHealth System: Results of a Randomised Trial. J Med Syst 2016;40:259. PMID: 27722974
18. Nicolucci A, Cercone S, Chiriatti A, Muscas F, Gensini G. A Randomized Trial on Home Telemonitoring for the Management of Metabolic and Cardiovascular Risk in Patients with Type 2 Diabetes. Diabetes Technol Ther 2015;17:563-570. PMID: 26154338
19. Tang PC, Overhage JM, Chan AS, Brown NL, Aghighi B, Entwistle MP, Hui SL, Hyde SM, Klieman LH, Mitchell CJ, Perkins AJ, Qureshi LS, Waltimyer TA, Winters LJ, Young CY. Online disease management of diabetes: engaging and motivating patients online with enhanced resources-diabetes (EMPOWER-D), a randomized controlled trial. J Am Med Inform Assoc 2013;20:526-534. PMID: 23171659
20. Kim HS, Jeong HS. A nurse short message service by cellular phone in type-2 diabetic patients for six months. J Clin Nurs 2007;16:1082-1087. PMID: 17518883
21. Kim CS, Park SY, Kang JG, Lee SJ, Ihm SH, Choi MG, Yoo HJ. Insulin dose titration system in diabetes patients using a short messaging service automatically produced by a knowledge matrix. Diabetes Technol Ther 2010;12:663-669. PMID: 20615108
22. Wakefield BJ, Koopman RJ, Keplinger LE, Bomar M, Bernt B, Johanning JL, Kruse RL, Davis JW, Wakefield DS, Mehr DR. Effect of home telemonitoring on glycemic and blood pressure control in primary care clinic patients with diabetes. Telemed J E Health 2014;20:199-205. PMID: 24404819
23. Stone RA, Rao RH, Sevick MA, Cheng C, Hough LJ, Macpherson DS, Franko CM, Anglin RA, Obrosky DS, Derubertis FR. Active care management supported by home telemonitoring in veterans with type 2 diabetes: the DiaTel randomized controlled trial. Diabetes Care 2010;33:478-484. PMID: 20009091
24. Pressman AR, Kinoshita L, Kirk S, Barbosa GM, Chou C, Minkoff J. A novel telemonitoring device for improving diabetes control: protocol and results from a randomized clinical trial. Telemed J E Health 2014;20:109-114. PMID: 24404816
25. Steventon A, Bardsley M, Doll H, Tuckey E, Newman SP. Effect of telehealth on glycaemic control: analysis of patients with type 2 diabetes in the Whole Systems Demonstrator cluster randomised trial. BMC Health Serv Res 2014;14:334. PMID: 25100190
26. Waki K, Fujita H, Uchimura Y, Omae K, Aramaki E, Kato S, Lee H, Kobayashi H, Kadowaki T, Ohe K. DialBetics: A Novel Smartphone-based Self-management Support System for Type 2 Diabetes Patients. Journal of Diabetes Science & Technology 2014;8:209. PMID: 24876569
27. Greenwood DA, Blozis SA, Young HM, Nesbitt TS, Quinn CC. Overcoming Clinical Inertia: A Randomized Clinical Trial of a Telehealth Remote Monitoring Intervention Using Paired Glucose Testing in Adults With Type 2 Diabetes. J Med Internet Res 2015;17:e178. PMID: 26199142

28. Weinstock RS, Teresi JA, Goland R, Izquierdo R, Palmas W, Eimicke JP, Ebner S, Shea S. Glycemic control and health disparities in older ethnically diverse underserved adults with diabetes: five-year results from the Informatics for Diabetes Education and Telemedicine (IDEATel) study. Diabetes Care 2011;34:274-279. PMID: 21270184
29. Liu CT YY, Lee TI, Li YC. Observations on online services for diabetes management. Diabetes Care 2005;28:2807-08:1-7. PMID: 16249564
30. Kim H-S, Song M-S. Technological intervention for obese patients with type 2 diabetes. Applied Nursing Research 2008;21:84-89. PMID: 18457747
31. Faridi Z, Liberti L, Shuval K, Northrup V, Ali A, Katz DL. Evaluating the impact of mobile telephone technology on type 2 diabetic patients' self-management: the NICHE pilot study. Journal of Evaluation in Clinical Practice 2008;14:465-469. PMID: 18373577
32. McMahon GT, Gomes HE, Hickson Hohne S, Hu TM-J, Levine BA, Conlin PR. Web-based care management in patients with poorly controlled diabetes. Diabetes care 2005;28:1624-1629. PMID: 15983311
33. Takenga C, Berndt R-D, Musongya O, Kitero J, Katoke R, Molo K, Kazingufu B, Meni M, Vikandy M, Takenga H. An ICT-Based Diabetes Management System Tested for Health Care Delivery in the African Context. International Journal of Telemedicine and Applications 2014;2014:437307-437310. PMID: 25136358
34. Yoo HJ, Park MS, Kim TN, Yang SJ, Cho GJ, Hwang TG, Baik SH, Choi DS, Park GH, Choi KM. A Ubiquitous Chronic Disease Care system using cellular phones and the internet. Diabetic medicine : a journal of the British Diabetic Association 2009;26:628-635. PMID: 19538239
35. Yoon K-H, Kim H-S. A short message service by cellular phone in type 2 diabetic patients for 12 months. Diabetes research and clinical practice 2008;79:256-261. PMID: 17988756
