# Supplementary material for: Effectiveness of Internet-Based Interventions on Glycemic Control in Patients With Type 2 Diabetes: Meta-Analysis of Randomized Controlled Trials
Source: J Med Internet Res. 2018 May 7;20(5):e172. doi: 10.2196/jmir.9133 (PMC5962831; doi:10.2196/jmir.9133)
Supplement: Multimedia Appendix 3 [file jmir_v20i5e172_app3.pdf]

Multimedia appendix 3: Characteristics of intervention group (IG) and control group (CG)

No., author, year	Intervention methods	Type of new media	Feedback
1. Zhou, P., 2014 [1]	Both groups: attended a 30-60 min educational session for diabetes self-management and nutrition. Everyone received a pamphlet of diabetes management and a schedule of diabetes courses.<br>IG: used telemedicine software to upload their blood glucose and other metabolic information at home at least every 2 weeks, and the researchers gave proper advices according to patients' key behaviors. They could log on internet at home to view the indicated homepage for self-education.<br>CG: without specific intervention, patients freely went to outpatient as usual.	Diabetes Telemedicine System (data transferring)	internet, short messages or telephone.
2. Orsama, A. L., 2013 [2]	IG: Patients used mobile application to upload diabetes-related parameters from home and received automatically generated, theory-based, health promotion-rich information, motivation, and behavioral skills feedback messages.<br>CG: standard medical care, including diabetes education, annual checkups, and diabetes guidance and education given by a doctor or nurse during patient-initiated visits to their health center.	mobile application Monica (data transferring)	automated messaging feedback
3. Avdal, E. U., 2011 [3]	IG: patients were monitored and educated via the web.<br>CG: patients were monitored and observed by the diabetes nurses in the polyclinic environment.<br>Education contents were formatted according to American Diabetes Association 2009 Criteria.	solely website. (data transferring)	through website
4. Noh, J. H., 2010 [4]	IG: Patients received training in eMOD usage and logged in to the eMOD system whenever it was convenient for them.<br>CG: Patients received diabetes educational books that had similar contents with the	eMOD system: a web-based ubiquitous information system for cellular phone and Internet users	not mentioned

No., author, year	Intervention methods	Type of new media	Feedback
	eMOD website. Both groups: patients visited their physicians every 2 months.	that provides diabetes self-management information.	
5. Tildesley, H. D., 2010 [5]	IG: uploaded SMBG readings every two weeks to a secure Website. They could also input medications, set up alarms, view a summary of readings, send messages to their endocrinologist and receive feedback. CG: kept a dairy of SMBG for every visit to their endocrinologist.	Internet-based Website, (data transferring)	through the Website
6. Cho, J. H., 2006 [6]	IG: Patients in the intervention group logged onto the Website at their convenience and uploaded their glucose levels, use of current medication, blood pressure and weight. In addition, patients recorded in the memo box any changes in their lifestyle and any questions or detailed information that the patient wished to discuss. CG: The patients in the control group used a conventional note-keeping record system.	Website (data transferring)	through the Website.
7. Kwon, H. S., 2004 [7]	IG: Patients in the intervention group logged onto the Website at their convenience and uploaded their glucose levels, drug medication, blood pressure and weight. In addition, patients recorded in the memo box any changes in their lifestyle and any questions or detailed information that the patient wished to discuss. CG: patients visited the center two or three times during the 3 months and received relative recommendations.	Website (data transferring)	through the Website.
8. Rodriguez-Idigoras, M. I., 2009 [8]	IG: patients received a glucometer and mobile phone. Patients sent blood glucose measurements to the call center in real-time via mobile phone. When blood glucose levels were not within normal range, the system sent an alarm to the call center, and previously established protocol interventions were implemented. Patients could also telephone their physician or the call center professional staff if they were not	Tele-assistance system: made up by patients' and physicians' mobile phones and the call center. (data transferring)	via mobile phone and web page.

No., author, year	Intervention methods	Type of new media	Feedback
	connected to the system.<br>CG: received regular follow-up at their healthcare center.		
9. Lim, S., 2016 [9]	Both groups: received pertinent diabetes education to standarize information level and diabetes management.<br>IG: The u-healthcare group was educated to use a specially designed glucometer and an activity monitor that automatically transferred test results to a hospital-based server and received automated messages.<br>CG: Routine care with SMBG	U-healthcare system. Websites and mobile phone (data transferring)	automated
10. Forjuoh, S. N., 2014 [10]	IG: patients input information of blood glucose, blood pressure, medication usage, physical activity and diary intake on PDA.<br>CG: did not receive any treatment other than usual clinical diabetes care, along with some publically available Texas Diabetes Council patient education materials.	personal digital assistant (PDA) (data transferring)	not mentioned
11. Glasgow, R. E., 2010 [11]	IG: patients used “My Path to Healthy Life” website and selected relative goals in terms of medication adherence, exercise, and food choices. They recorded progress in the website and received immediate feedback on success meeting their goals. They also received periodic telephone promptings.<br>CG: usual care.	website	not mentioned
12. Quinn, C. C., 2011 [12]	IG1: entered data into a mobile application and received automated, real-time educational, behavioral, and motivational messaging. Patient web portal consisted of a messaging center, personal health record and learning library. Healthcare provider portal allowed provider to view unanalyzed patients' data.<br>IG2: in addition to intervention in IG1, provider received quarterly reports that summarized patients’ relevant data.	website and mobile application (data transferring)	automated messaging feedback

No., author, year	Intervention methods	Type of new media	Feedback
	CG: usual care.		
13. Bujnowska-Fedak, 2011 [13]	IG: patients received an in-home wireless glucose monitor and transmitter, downloaded data to their PC and sent to doctors via Internet. Glucose value exceeded a set range would trigger an audio-video alarm at the clinic and generate a text message to the cellular phone of caregiver (GP) to provide urgent medical attention.<br>CG: followed the usual arrangement they had before the study.	computer (data transferring)	not mentioned
14. Hsu, W. C., 2016 [14]	IG: received care through cloud-based diabetes management program where regular communications about glycemic control and insulin doses were conducted via patient self-tracking tools, shared decision-making interfaces, secure text messages, and virtual visits (audio, video and shared screen control).<br>CG: received standard face-to-face care and phone follow-up as needed in the tertiary center.	tablet computer application (data transferring)	virtual visits and secure text messages.
15. Dario, C., 2016 [15]	IG: Patients were equipped at home with a glucometer and a gateway for data transmission to a Regional eHealth Center (ReHC). The ReHC processed clinical data automatically to identify possible alarm values and informed clinicians.<br>CG: had access to usual care and shared the paper log books of glucometer tests with GP during planned visits.	data transferring equipment	not mentioned
16. Torbjornsen, A., 2014 [16]	IG: participants were given a mobile phone for 1 year, which provided access to Few Touch Application diabetes diary that recorded 5 elements: blood glucose, food habits, physical activity, personal goal setting, and a look-up system for diabetes information.<br>CG: received usual care according to the Norwegian clinical guidelines.	mobile application FTA and data transferring equipment	secured text messaging, telephone

No., author, year	Intervention methods	Type of new media	Feedback
17. Kardas, P., 2016 [17]	IG: patients were equipped with COMMODITY12 system, composed of smart phone and wirelessly connected sensors.<br>CG: standard care	mobile phone (data transferring)	not mentioned
18. Nicolucci, A., 2015 [18]	IG: glucometer, weight scale and sphygmomanometer connected via bluetooth to a hub could transmit information in real time. Patients could receive reminders, notifications and warning messages (text message, e-mail or telephone). Doctors could access patients' data from any site.<br>CG: usual care.	data transferring equipment	telephone
19. Tang, P. C., 2013 [19]	IG: The intervention included: wirelessly uploaded home glucometer readings with graphical feedback; comprehensive patient-specific diabetes summary status report; nutrition and exercise logs; insulin record; online messaging with the patient’s health team; nurse care manager and dietitian providing advice and medication management and personalized text and video educational ‘nuggets’ dispensed electronically by the care team.<br>CG: usual care	smart phone and website (data transferring)	secure messaging
20. Kim, H. S., 2007 [20]	IG: Patients were asked to access a website by using a cellular phone or to wiring the Internet and input their blood glucose levels every day. Participants were sent the optimal recommendations using SMS by both cellular phone and the Internet weekly.<br>CG: met the endocrinologist specialist once or twice during the six months and were provided with recommendations about medication, medication dosage and lifestyle modification as customary in the outpatient clinic.	mobile phone and website (data transferring)	short message service by cellular phone or internet
21. Kim, C. S., 2010 [21]	IG: a glucometer was connected to patients’ cellular phone which could automatically send glucose information to their personal data sheet on a website. The	mobile phone	automated messages

No., author, year	Intervention methods	Type of new media	Feedback
	system automatically sent messages to patients to adjust insulin dose.<br>CG: Patients received conventional titration scheme and self-adjusted their basal insulin according to daily self-monitored capillary FBG measurements.		
22. Wakefield, B. J., 2014 [22]	IG: patients had BG meter and BP monitor. Self-measured BP and BG data could be transferred directly over either an analog phone line or a personal computer with Internet access to a secure website. Participants with access to the Internet were able to track and monitor their progress on the patient section of the Web site.<br>CG: patients were instructed to record BG and BP readings and bring their records to their clinic visits.	data transferring equipment	telephone
23. Stone, R. A., 2010 [23]	Both group received 2-h educational session for diabetes self-management and nutrition.<br>IG: used telemonitoring device which permitted continuous home messaging with reminders and education; ongoing monitoring of SMBG, BP, and weight; and daily transmission of these data to study providers via a secure network. Nurses also called participants monthly to provide individualized self-management counseling tailored to specific issues.<br>CG: received monthly telephone calls from the study diabetes nurse educator regarding general health conditions, status of glycemic control, BP, and weight from daily logs maintained by the participants	home telemonitoring devices (allowed data transmission)	telephone
24. Pressman, A. R., 2014 [24]	IG: used telemetry device at home to transmit glucose levels, BP readings, and weight measurements to the diabetes care manager weekly and received usual telephone visits as described below.<br>CG: received usual care management program (an initial in-person office visit	telemetry device (data transferring)	telephone, telemetry device

No., author, year	Intervention methods	Type of new media	Feedback
	followed by telephone visits: weekly telephone calls for one month followed by monthly calls).		
25. Steventon, A., 2014 [25]	IG: used telehealth equipment which included a base unit (freestanding or a television set top box), a glucometer and a BP monitor. The telehealth equipment reminded participants to take physiological measurements and sent them symptom questions and educational messages.<br>CG: They received usual care for the study sites, which excluded telehealth but for some patients included self-monitoring of blood glucose	telehealth equipment (data transferring)	via the telehealth base unit or set top box.
26. Waki, K., MD, 2014 [26]	IG: patients received a mobile phone, glucometer and BP monitor. Measurements could be transmitted to healthcare provider.<br>CG: continued their selfcare regimen.	mobile phone and data transferring equipment	not mentioned
27. Greenwood, D. A., 2015 [27]	IG: patients received a tablet computer which could automatically transmit glucose data and facilitated a complete feedback loop to educate participants, analyze actionable glucose data and provide feedback.<br>CG: patients received diabetes education booklets and referral for formal diabetes education as needed. This group continued to receive nurse care coordination including reminders for A1c and health maintenance exams sent by postal mail.	tablet computer and data transferring equipment	virtual visits (secure messaging) and telephone.
28. Weinstock, R. S., 2011 [28]	IG: Telemedicine subjects received a home telemedicine unit to videoconference with a diabetes educator for self-management education, review of transmitted home blood glucose and blood pressure measurements, individualized goal-setting, and access to educational web pages.<br>CG: usual care.	telemedicine unit with data transferring function (computer-based)	videoconferencing
29. Liu, C.T.,	IG: POEM system allowed analysis of laboratory results and access to records	combined website and mobile	not mentioned

No., author, year	Intervention methods	Type of new media	Feedback
2005	online. E-mails and SMS via cell phones was used to provide reminders to patients<br>CG: usual care.		
30. Kim, H.S., 2008	IG: Web-based program allowing shared access of electronic medical records. Patients sent blood glucose data and drug information to the website. A researcher reviewed results weekly and optimal recommendations were sent via cell phone and internet.<br>CG: visit primary care doctor and take medicine as usual	combined website and mobile	via cell phone and internet from researcher
31. Faridi, Z., 2008	IG: Subjects were required to upload data onto the NICHE server once daily. They received tailored messages via mobile phone based on the uploaded data.<br>CG: subjects continued with standard diabetes self-management and tracked their step count using a pedometer.	mobile-based	tailored messages via mobile phone
32. McMahon, G.T., 2005	IG: Recommendations were provided based on blood pressure and blood glucose data uploaded by patients. The patients communicated with the care manager via the IM and telephone. The website also contained web-enabled diabetes educational modules and had links to other web-based diabetes resources.<br>CG: usual care.	web-based	feedbacks from doctors
33. Takenga, C., 2014	IG: Patients entered data into Mobil Diab and received recommendations and feedbacks from doctors<br>CG: usual care.	combined website and mobile	feedbacks from doctors
34. Yoo, H.J., 2009	IG: Patients entered data into a mobile app and received automated feedbacks immediately<br>CG: usual care.	mobile-based	automated

No., author, year	Intervention methods	Type of new media	Feedback
35. Yoon, K.H., 2008	IG: Patients in the intervention group were asked to access a website by using a cellular phone or to wiring the Internet and input their blood glucose levels weekly. Participants were sent the optimal recommendations by both cellular phone and the Internet weekly.<br>CG: usual care.	combined website and mobile	by both cellular phone and the Internet

Reference

1. Zhou P, Xu L, Liu X, Huang J, Xu W, Chen W. Web-based telemedicine for management of type 2 diabetes through glucose uploads: a randomized controlled trial. Int J Clin Exp Pathol 2014;7:8848-8854. PMID: 25674254
2. Orsama AL, Lahteenmaki J, Harno K, Kulju M, Wintergerst E, Schachner H, Stenger P, Leppanen J, Kaijanranta H, Salaspuro V, Fisher WA. Active assistance technology reduces glycosylated hemoglobin and weight in individuals with type 2 diabetes: results of a theory-based randomized trial. Diabetes Technol Ther 2013;15:662-669. PMID: 23844570
3. Avdal EU, Kizilci S, Demirel N. The effects of web-based diabetes education on diabetes care results: a randomized control study. Comput Inform Nurs 2011;29:101-106. PMID: 21099675
4. Noh JH, Cho YJ, Nam HW, Kim JH, Kim DJ, Yoo HS, Kwon YW, Woo MH, Cho JW, Hong MH, Yoo JH, Gu MJ, Kim SA, An KE, Jang SM, Kim EK, Yoo HJ. Web-based comprehensive information system for self-management of diabetes mellitus. Diabetes Technol Ther 2010;12:333-337. PMID: 20388042
5. Tildesley HD, Mazanderani AB, Ross SA. Effect of Internet therapeutic intervention on A1C levels in patients with type 2 diabetes treated with insulin. Diabetes Care 2010;33:1738-1740. PMID: 20668152
6. Cho JH, Chang SA, Kwon HS, Choi YH, Ko SH, Moon SD, Yoo SJ, Song KH, Son HS, Kim HS, Lee WC, Cha BY, Son HY, Yoon KH. Long-term effect of the Internet-based glucose monitoring system on HbA1c reduction and glucose stability: a 30-month follow-up study for diabetes management with a ubiquitous medical care system. Diabetes Care 2006;29:2625-2631. PMID: 17130195
7. Kwon HS, Cho JH, Kim HS, Song BR, Ko SH, Lee JM, Kim SR, Chang SA, Kim HS, Cha BY, Lee KW, Son HY, Lee JH, Lee WC, Yoon KH.

Establishment of blood glucose monitoring system using the internet. Diabetes Care 2004;27:478-483. PMID: 14747232
8. Rodriguez-Idigoras MI, Sepulveda-Munoz J, Sanchez-Garrido-Escudero R, Martinez-Gonzalez JL, Escolar-Castello JL, Paniagua-Gomez IM, Bernal-Lopez R, Fuentes-Simon MV, Garofano-Serrano D. Telemedicine influence on the follow-up of type 2 diabetes patients. Diabetes Technol Ther 2009;11:431-437. PMID: 19580356
9. Lim S, Kang SM, Kim KM, Moon JH, Choi SH, Hwang H, Jung HS, Park KS, Ryu JO, Jang HC. Multifactorial intervention in diabetes care using real-time monitoring and tailored feedback in type 2 diabetes. Acta Diabetol 2016;53:189-198. PMID: 25936739
10. Forjuoh SN, Bolin JN, Huber JC, Jr., Vuong AM, Adepoju OE, Helduser JW, Begaye DS, Robertson A, Moudouni DM, Bonner TJ, McLeroy KR, Ory MG. Behavioral and technological interventions targeting glycemic control in a racially/ethnically diverse population: a randomized controlled trial. BMC Public Health 2014;14:71. PMID: 24450992
11. Glasgow RE, Kurz D, King D, Dickman JM, Faber AJ, Halterman E, Wooley T, Toobert DJ, Strycker LA, Estabrooks PA, Osuna D, Ritzwoller D. Outcomes of minimal and moderate support versions of an internet-based diabetes self-management support program. J Gen Intern Med 2010;25:1315-1322. PMID: 20714820
12. Quinn CC, Shardell MD, Terrin ML, Barr EA, Ballew SH, Gruber-Baldini AL. Cluster-randomized trial of a mobile phone personalized behavioral intervention for blood glucose control. Diabetes Care 2011;34:1934-1942. PMID: 21788632
13. Bujnowska-Fedak MM, Puchala E, Steciwko A. The impact of telehome care on health status and quality of life among patients with diabetes in a primary care setting in Poland. Telemed J E Health 2011;17:153-163. PMID: 21375410
14. Hsu WC, Lau KH, Huang R, Ghiloni S, Le H, Gilroy S, Abrahamson M, Moore J. Utilization of a Cloud-Based Diabetes Management Program for Insulin Initiation and Titration Enables Collaborative Decision Making Between Healthcare Providers and Patients. Diabetes Technol Ther 2016;18:59-67. PMID: 26645932
15. Dario C, Toffanin R, Calcaterra F, Saccavini C, Stafylas P, Mancin S, Vio E. Telemonitoring of Type 2 Diabetes Mellitus in Italy. Telemedicine journal and e-health : the official journal of the American Telemedicine Association 2016. PMID: 27379995
16. Torbjornsen A, Jenum AK, Smastuen MC, Arsand E, Holmen H, Wahl AK, Ribu L. A Low-Intensity Mobile Health Intervention With and Without Health Counseling for Persons With Type 2 Diabetes, Part 1: Baseline and Short-Term Results From a Randomized Controlled Trial in the Norwegian Part of RENEWING HEALTH. JMIR Mhealth Uhealth 2014;2:e52. PMID: 25499592
17. Kardas P, Lewandowski K, Bromuri S. Type 2 Diabetes Patients Benefit from the COMODITY12 mHealth System: Results of a Randomised Trial. J

Med Syst 2016;40:259. PMID: 27722974
18. Nicolucci A, Cercone S, Chiriatti A, Muscas F, Gensini G. A Randomized Trial on Home Telemonitoring for the Management of Metabolic and Cardiovascular Risk in Patients with Type 2 Diabetes. Diabetes Technol Ther 2015;17:563-570. PMID: 26154338
19. Tang PC, Overhage JM, Chan AS, Brown NL, Aghighi B, Entwistle MP, Hui SL, Hyde SM, Klieman LH, Mitchell CJ, Perkins AJ, Qureshi LS, Waltimyer TA, Winters LJ, Young CY. Online disease management of diabetes: engaging and motivating patients online with enhanced resources-diabetes (EMPOWER-D), a randomized controlled trial. J Am Med Inform Assoc 2013;20:526-534. PMID: 23171659
20. Kim HS, Jeong HS. A nurse short message service by cellular phone in type-2 diabetic patients for six months. J Clin Nurs 2007;16:1082-1087. PMID: 17518883
21. Kim CS, Park SY, Kang JG, Lee SJ, Ihm SH, Choi MG, Yoo HJ. Insulin dose titration system in diabetes patients using a short messaging service automatically produced by a knowledge matrix. Diabetes Technol Ther 2010;12:663-669. PMID: 20615108
22. Wakefield BJ, Koopman RJ, Keplinger LE, Bomar M, Bernt B, Johanning JL, Kruse RL, Davis JW, Wakefield DS, Mehr DR. Effect of home telemonitoring on glycemic and blood pressure control in primary care clinic patients with diabetes. Telemed J E Health 2014;20:199-205. PMID: 24404819
23. Stone RA, Rao RH, Sevick MA, Cheng C, Hough LJ, Macpherson DS, Franko CM, Anglin RA, Obrosky DS, Derubertis FR. Active care management supported by home telemonitoring in veterans with type 2 diabetes: the DiaTel randomized controlled trial. Diabetes Care 2010;33:478-484. PMID: 20009091
24. Pressman AR, Kinoshita L, Kirk S, Barbosa GM, Chou C, Minkoff J. A novel telemonitoring device for improving diabetes control: protocol and results from a randomized clinical trial. Telemed J E Health 2014;20:109-114. PMID: 24404816
25. Steventon A, Bardsley M, Doll H, Tuckey E, Newman SP. Effect of telehealth on glycaemic control: analysis of patients with type 2 diabetes in the Whole Systems Demonstrator cluster randomised trial. BMC Health Serv Res 2014;14:334. PMID: 25100190
26. Waki K, Fujita H, Uchimura Y, Omae K, Aramaki E, Kato S, Lee H, Kobayashi H, Kadowaki T, Ohe K. DialBetics: A Novel Smartphone-based Self-management Support System for Type 2 Diabetes Patients. Journal of Diabetes Science & Technology 2014;8:209. PMID: 24876569
27. Greenwood DA, Blozis SA, Young HM, Nesbitt TS, Quinn CC. Overcoming Clinical Inertia: A Randomized Clinical Trial of a Telehealth Remote Monitoring Intervention Using Paired Glucose Testing in Adults With Type 2 Diabetes. J Med Internet Res 2015;17:e178. PMID: 26199142
28. Weinstock RS, Teresi JA, Goland R, Izquierdo R, Palmas W, Eimicke JP, Ebner S, Shea S. Glycemic control and health disparities in older ethnically diverse underserved adults with diabetes: five-year results from the Informatics for Diabetes Education and Telemedicine (IDEATel) study. Diabetes Care 2011;34:274-279. PMID: 21270184

29. Liu CT YY, Lee TI, Li YC. Observations on online services for diabetes management. Diabetes Care 2005;28:2807-08:1-7. PMID: 16249564
30. Kim H-S, Song M-S. Technological intervention for obese patients with type 2 diabetes. Applied Nursing Research 2008;21:84-89. PMID: 18457747
31. Faridi Z, Liberti L, Shuval K, Northrup V, Ali A, Katz DL. Evaluating the impact of mobile telephone technology on type 2 diabetic patients' self-management: the NICHE pilot study. Journal of Evaluation in Clinical Practice 2008;14:465-469. PMID: 18373577
32. McMahon GT, Gomes HE, Hickson Hohne S, Hu TM-J, Levine BA, Conlin PR. Web-based care management in patients with poorly controlled diabetes. Diabetes care 2005;28:1624-1629. PMID: 15983311
33. Takenga C, Berndt R-D, Musongya O, Kitero J, Katoke R, Molo K, Kazingufu B, Meni M, Vikandy M, Takenga H. An ICT-Based Diabetes Management System Tested for Health Care Delivery in the African Context. International Journal of Telemedicine and Applications 2014;2014:437307-437310. PMID: 25136358
34. Yoo HJ, Park MS, Kim TN, Yang SJ, Cho GJ, Hwang TG, Baik SH, Choi DS, Park GH, Choi KM. A Ubiquitous Chronic Disease Care system using cellular phones and the internet. Diabetic medicine : a journal of the British Diabetic Association 2009;26:628-635. PMID: 19538239
35. Yoon K-H, Kim H-S. A short message service by cellular phone in type 2 diabetic patients for 12 months. Diabetes research and clinical practice 2008;79:256-261. PMID: 17988756
