# Supplementary material for: Effectiveness of Internet-Based Interventions on Glycemic Control in Patients With Type 2 Diabetes: Meta-Analysis of Randomized Controlled Trials
Source: J Med Internet Res. 2018 May 7;20(5):e172. doi: 10.2196/jmir.9133 (PMC5962831; doi:10.2196/jmir.9133)
Supplement: Multimedia Appendix 4 [file jmir_v20i5e172_app4.pdf]

Multimedia appendix 4. Publication bias

According to Figure 1, Publication bias was not detected in Begg's test as z =1.76 < 1.96 and p = 0.079 > 0.05. However, publication bias was detected in Egger's test as p = 0.02 < 0.05. As a result, we further conducted the trim and fill method to account for publication bias. [1-2] In this meta-analysis, as studies with positive or large effect sizes were likely to be suppressed, flip option was used in trim and fill method.

In the trim and fill method, although the strength of intervention was slightly attenuated, the pooled analysis incorporating the hypothetical studies continued to show a statistically significant positive effect of Internet-based interventions (WMD: -0.332, 95% CI: [-0.456, -0.209], p < 0.001), which suggested that the mean difference of HbA1c between intervention and control group was not an artifact of unpublished negative studies, but the effect of Internet-based interventions. However, the possibility was not altogether excluded by this method.

Figure 1. Publication bias in Begg's and Egger's test

```
Tests for Publication Bias

Begg's Test

  adj. Kendall's Score (P-Q) =     -130
          Std. Dev. of Score =    73.42
           Number of Studies =       36
                          z  =    -1.77
                    Pr > |z| =    0.077
                          z  =     1.76 (continuity corrected)
                    Pr > |z| =    0.079 (continuity corrected)

Egger's test
```

Std_Eff	Coef.	Std. Err.	t	P>|t|	[95% Conf.	Interval]
slope	-.085573	.1274844	-0.67	0.507	-.3446525	.1735065
bias	-1.534634	.6291014	-2.44	0.020	-2.813122	-.2561465

Figure 2. Result of the trim and fill method

```
Meta-analysis

       |  Pooled      95% CI         Asymptotic      No. of
Method |     Est   Lower   Upper  z_value  p_value   studies
-------+----------------------------------------------------
Fixed  |  -0.372  -0.438  -0.305  -11.001    0.000     36
Random |  -0.426  -0.540  -0.312   -7.323    0.000

Test for heterogeneity: Q= 87.827 on 35 degrees of freedom (p= 0.000)
Moment-based estimate of between studies variance =  0.064

Trimming estimator: Linear
Meta-analysis type: Random-effects model

iteration |  estimate    Tn    # to trim     diff
----------+--------------------------------------
    1     |   -0.426    365        2          666
    2     |   -0.366    415        5          100
    3     |   -0.343    448        6           66
    4     |   -0.335    456        7           16
    5     |   -0.325    464        7           16
    6     |   -0.325    464        7            0

Filled
Meta-analysis

       |  Pooled      95% CI         Asymptotic      No. of
Method |     Est   Lower   Upper  z_value  p_value   studies
-------+----------------------------------------------------
Fixed  |  -0.317  -0.381  -0.253   -9.759    0.000     43
Random |  -0.332  -0.456  -0.209   -5.265    0.000

Test for heterogeneity: Q= 134.904 on 42 degrees of freedom (p= 0.000)
Moment-based estimate of between studies variance =  0.103
```

Figure 3. Funnel plot without and with trim and fill

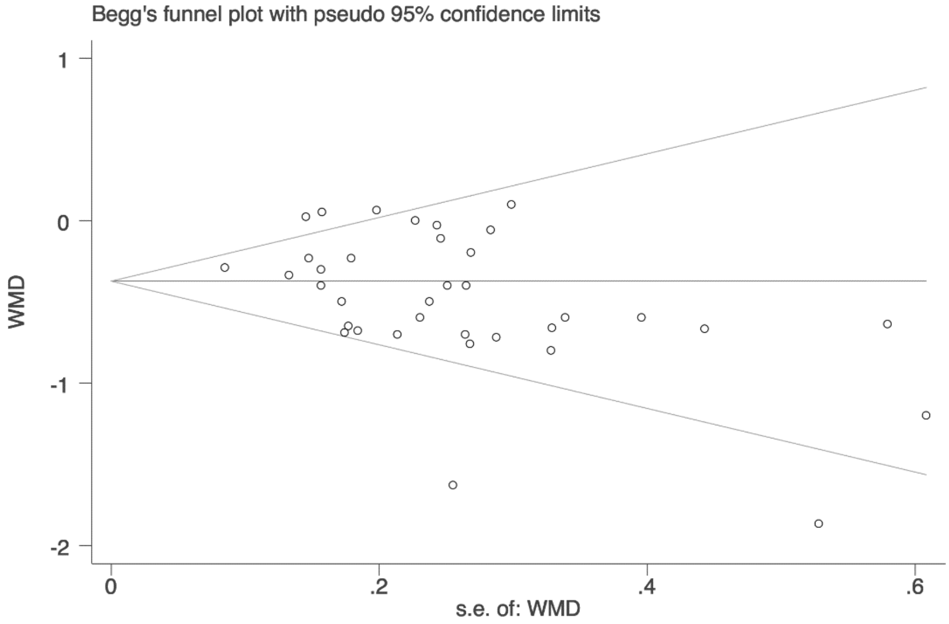

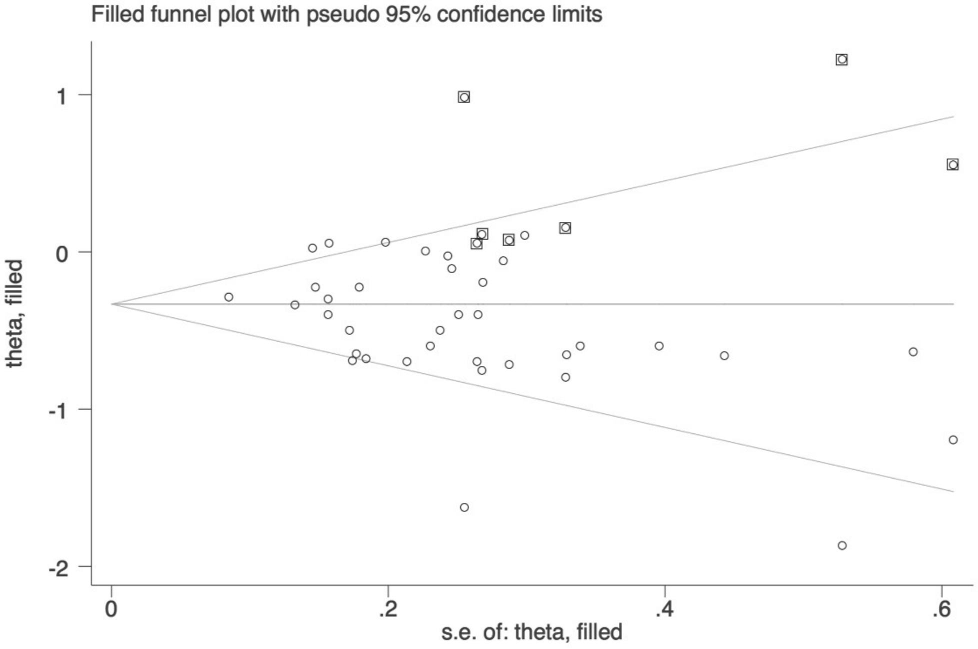

[1] Duval, S., & Tweedie, R. (2000). Trim and fill: A simple funnel-plot-based method of testing and adjusting for publication bias in meta-analysis. *Biometrics*, *56*(2), 455–463.

[2] Willi, C., Bodenmann, P., Ghali, W. A., Faris, P. D., & Cornuz, J. (2007). Active smoking and the risk of type 2 diabetes: a systematic review and meta-analysis. *Jama*, *298*(22), 2654–2664. http://doi.org/10.1001/jama.298.22.2654
