# Supplementary material for: Effectiveness of Internet-Based Interventions on Glycemic Control in Patients With Type 2 Diabetes: Meta-Analysis of Randomized Controlled Trials
Source: J Med Internet Res. 2018 May 7;20(5):e172. doi: 10.2196/jmir.9133 (PMC5962831; doi:10.2196/jmir.9133)
Supplement: Multimedia Appendix 5 [file jmir_v20i5e172_app5.pdf]

Multimedia appendix 5: risk of bias

The following figures described the risk of bias in each study. Although all studies were randomized and therefore the risk of bias was lower in this domain, only 18 clearly stated the process of random sequence generation [1-18]. The risk of bias due to allocation concealment was high in one study whose allocation process was not concealed based on its description [14]. Due to the nature of intervention, it was not possible to blind either participants or researchers. Therefore, all studies were at high risk in blinding of participants and personnel. 5 studies reported blinding of outcome assessment [6, 8, 19-21] and 2 studies clearly stated that outcome assessment was not blinded [11, 14]. All studies were free from incomplete outcome data and all had a low risk of reporting bias except two which only reported systolic blood pressure and omitted diastolic blood pressure [21, 22]. Other bias was unclear in all studies.

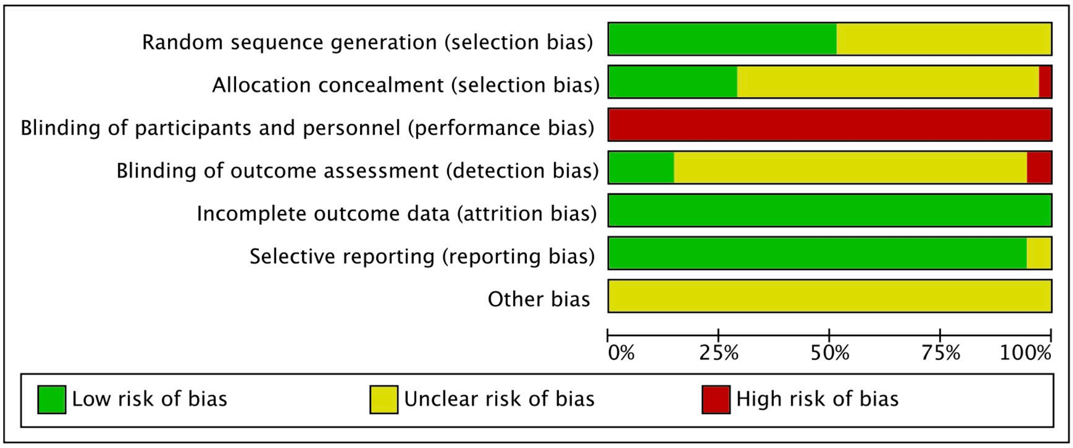

(a) Risk of bias graph: review authors' judgements about each risk of bias item presented as percentages across all included studies.

	Random sequence generation (selection bias)	Allocation concealment (selection bias)	Blinding of participants and personnel (performance bias)	Blinding of outcome assessment (detection bias)	Incomplete outcome data (attrition bias)	Selective reporting (reporting bias)	Other bias
Avdal 2011	+	+	-	?	+	+	?
Bujnowska-Fedak 2011	?	?	-	?	+	+	?
Cho, J. H. 2006	?	?	-	?	+	+	?
Dario 2016	+	+	-	?	+	+	?
Faridi, Z. 2008	?	?	-	?	+	+	?
Forjuoh, S. N. 2014	+	?	-	?	+	+	?
Glasgow 2010	+	?	-	?	+	+	?
Greenwood, D. A. 2015	+	-	-	-	+	+	?
Hsu, W. C. 2016	?	?	-	?	+	+	?
Kardas, P. 2016	?	?	-	?	+	+	?
Kayo Waki, MD 2104	+	?	-	?	+	+	?
Kim, C. S. 2010	?	+	-	?	+	+	?
Kim, H. S. 2007	+	?	-	?	+	+	?
Kim, H.S. 2008	+	?	-	?	+	+	?
Kwon, H. S. 2004	?	?	-	?	+	+	?
Lim, S. 2016	?	?	-	?	+	+	?
Liu, C.T. 2005	?	?	-	?	+	+	?
McMahon, G.T. 2005	+	+	-	?	+	+	?
Nicolucci, A. 2015	?	+	-	?	+	+	?
Noh 2010	?	?	-	?	+	+	?
Orsama 2013	+	?	-	?	+	+	?
Pressman, A. R 2014	?	?	-	+	+	?	?
Quinn, C. C. 2011	+	+	-	+	+	+	?
Rodriguez-Idigoras, M. I. 2009	+	+	-	?	+	+	?
Steventon, A. 2014	+	+	-	?	+	+	?
Stone, R. A. 2010	?	?	-	+	+	+	?
Takenga, C. 2014	?	?	-	?	+	+	?
Tang, P. C. 2013	+	?	-	+	+	+	?
Tildesley, H. D. 2010	+	?	-	?	+	+	?
Torbjornsen, A.2 2014	+	+	-	-	+	+	?
Wakefield, B. J. 2014	?	+	-	?	+	?	?
Weinstock, R. S. 2011	?	?	-	+	+	+	?
Yoo, H.J. 2009	?	?	-	?	+	+	?
Yoon, K.H. 2008	+	?	-	?	+	+	?
Zhou 2014	+	?	-	?	+	+	?

(b) Risk of bias summary: review authors' judgements about each risk of bias item for each included study.

Reference

[1] Kim HS, Jeong HS (2007) A nurse short message service by cellular phone in type-2 diabetic patients for six months. Journal of clinical nursing 16: 1082-1087

[2] Rodriguez-Idigoras MI, Sepulveda-Munoz J, Sanchez-Garrido-Escudero R, et al. (2009) Telemedicine influence on the follow-up of type 2 diabetes patients. Diabetes technology & therapeutics 11: 431-437

[3] Glasgow RE, Kurz D, King D, et al. (2010) Outcomes of minimal and moderate support versions of an internet-based diabetes self-management support program. J Gen Intern Med 25: 1315-1322

[4] Tildesley HD, Mazanderani AB, Ross SA (2010) Effect of Internet therapeutic intervention on A1C levels in patients with type 2 diabetes treated with insulin. Diabetes Care 33: 1738-1740

[5] Avdal EU, Kizilci S, Demirel N (2011) The effects of web-based diabetes education on diabetes care results: a randomized control study. Comput Inform Nurs 29: 101-106

[6] Quinn CC, Shardell MD, Terrin ML, Barr EA, Ballew SH, Gruber-Baldini AL (2011) Cluster-randomized trial of a mobile phone personalized behavioral intervention for blood glucose control. Diabetes care 34: 1934-1942

[7] Orsama AL, Lahteenmaki J, Harno K, et al. (2013) Active assistance technology reduces glycosylated hemoglobin and weight in individuals with type 2 diabetes: results of a theory-based randomized trial. Diabetes Technol Ther 15: 662-669

[8] Tang PC, Overhage JM, Chan AS, et al. (2013) Online disease management of diabetes: engaging and motivating patients online with enhanced resources-diabetes (EMPOWER-D), a randomized controlled trial. Journal of the American Medical Informatics Association : JAMIA 20: 526-534

[9] Forjuoh SN, Bolin JN, Huber JC, Jr., et al. (2014) Behavioral and technological interventions targeting glycemic control in a racially/ethnically diverse population: a randomized controlled trial. BMC public health 14: 71

[10] Steventon A, Bardsley M, Doll H, Tuckey E, Newman SP (2014) Effect of telehealth on glycaemic control: analysis of patients with type 2 diabetes in the Whole Systems Demonstrator cluster randomised trial. BMC health services research 14: 334

[11] Torbjornsen A, Jenum AK, Smastuen MC, et al. (2014) A Low-Intensity Mobile Health Intervention With and Without Health Counseling for Persons With Type 2 Diabetes, Part 1: Baseline and Short-Term Results From a Randomized Controlled Trial in the Norwegian Part of RENEWING HEALTH. JMIR Mhealth Uhealth 2: e52

[12] Waki K, Fujita H, Uchimura Y, et al. (2014) DialBetics: A Novel Smartphone-based Self-management Support System for Type 2 Diabetes Patients. Journal of Diabetes Science & Technology 8: 209

[13] Zhou P, Xu L, Liu X, Huang J, Xu W, Chen W (2014) Web-based telemedicine for management of type 2 diabetes through glucose uploads: a randomized controlled trial. International journal of clinical and experimental pathology 7: 8848-8854
[14] Greenwood DA, Blozis SA, Young HM, Nesbitt TS, Quinn CC (2015) Overcoming Clinical Inertia: A Randomized Clinical Trial of a Telehealth Remote Monitoring Intervention Using Paired Glucose Testing in Adults With Type 2 Diabetes. Journal of medical Internet research 17: e178
[15] Dario C, Toffanin R, Calcaterra F, et al. (2016) Telemonitoring of Type 2 Diabetes Mellitus in Italy. Telemedicine journal and e-health : the official journal of the American Telemedicine Association
[16] Kim H-S, Song M-S (2008) Technological intervention for obese patients with type 2 diabetes. Applied Nursing Research 21: 84-89
[17] McMahon GT, Gomes HE, Hickson Hohne S, Hu TM-J, Levine BA, Conlin PR (2005) Web-based care management in patients with poorly controlled diabetes. Diabetes care 28: 1624-1629
[18] Yoon K-H, Kim H-S (2008) A short message service by cellular phone in type 2 diabetic patients for 12 months. Diabetes research and clinical practice 79: 256-261
[19] Stone RA, Rao RH, Sevick MA, et al. (2010) Active care management supported by home telemonitoring in veterans with type 2 diabetes: the DiaTel randomized controlled trial. Diabetes care 33: 478-484
[20] Weinstock RS, Teresi JA, Goland R, et al. (2011) Glycemic control and health disparities in older ethnically diverse underserved adults with diabetes: five-year results from the Informatics for Diabetes Education and Telemedicine (IDEATel) study. Diabetes care 34: 274-279
[21] Pressman AR, Kinoshita L, Kirk S, Barbosa GM, Chou C, Minkoff J (2014) A novel telemonitoring device for improving diabetes control: protocol and results from a randomized clinical trial. Telemedicine journal and e-health : the official journal of the American Telemedicine Association 20: 109-114
[22] Wakefield BJ, Koopman RJ, Keplinger LE, et al. (2014) Effect of home telemonitoring on glycemic and blood pressure control in primary care clinic patients with diabetes. Telemedicine journal and e-health : the official journal of the American Telemedicine Association 20: 199-205
