# Supplementary material for: Effectiveness of Internet-Based Interventions on Glycemic Control in Patients With Type 2 Diabetes: Meta-Analysis of Randomized Controlled Trials
Source: J Med Internet Res. 2018 May 7;20(5):e172. doi: 10.2196/jmir.9133 (PMC5962831; doi:10.2196/jmir.9133)

Multimedia appendix 6: sensitivity analysis

We performed sensitivity analysis by omitting individual studies in sequence to evaluate the effect of individual studies on the pooled results. Results showed that sensitivity of the 35 studies was low as omission of individual studies did not overturn or change the pooled results significantly.

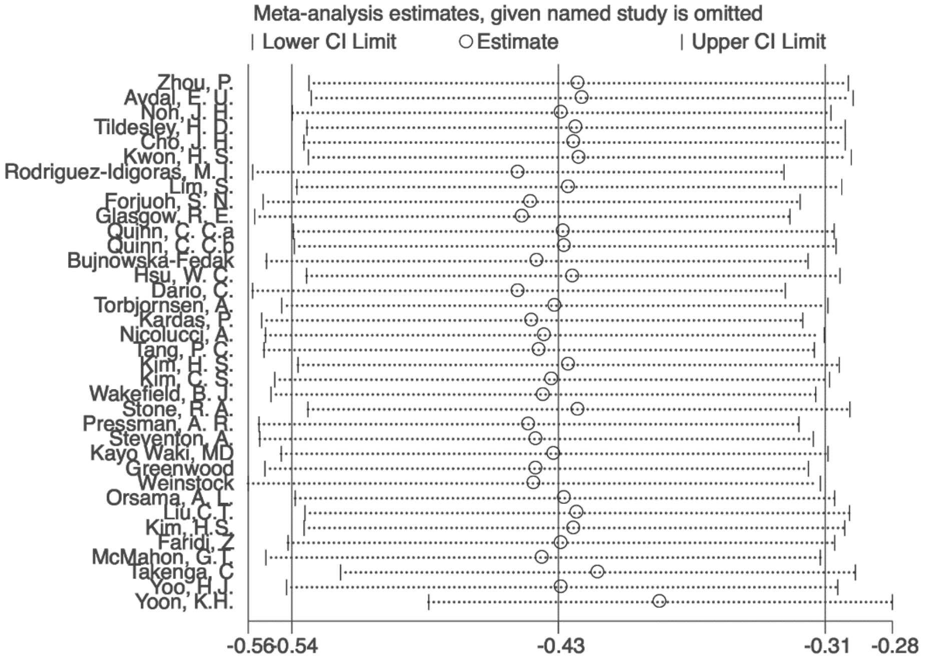

Supplement: Multimedia Appendix 6 [file jmir_v20i5e172_app6.pdf]
