# Supplementary material for: Effectiveness of Internet-Based Interventions on Glycemic Control in Patients With Type 2 Diabetes: Meta-Analysis of Randomized Controlled Trials
Source: J Med Internet Res. 2018 May 7;20(5):e172. doi: 10.2196/jmir.9133 (PMC5962831; doi:10.2196/jmir.9133)
Supplement: Multimedia Appendix 7 [file jmir_v20i5e172_app7.pdf]

Multimedia appendix 7: HbA1c value and related data of intervention group (IG) and control group (CG) at baseline and endpoint

study	Baseline HbA1c % (mmol/mol)						Intervention duration	Post-intervention HbA1c % (mmol/mol)					
	CG			IG				CG			IG		
	N	Mean	SD	*N*	Mean	SD		*N*	Mean	SD	*N*	Mean	SD
1. Zhou, P., 2014 [1]	55	8.22 (66)	1.58 (17.3)	53	8.44 (69)	1.58 (17.3)	3 months	55	7.6 (60)	1.57 (17.2)	53	6.84 (51)	1.2 (13.1)
2. Orsama, A. L., 2013 [2]	24	7.09 (54)	1.51 (16.5)	24	6.86 (51)	1.56 (17.1)	10 months	24	7.13 (54)	1.51 (16.5)	24	6.46 (47)	1.56 (17.1)
3. Avdal, E. U., 2011 [3]	61	8.14 (65)	0.99 (10.8)	61	8.01 (64)	0.97 (10.6)	6 months	61	8.19 (66)	1.15 (12.6)	61	7.50 (58)	0.73 (8.0)
4. Noh, J. H., 2010 [4]	20	8.6 (70)	1.2 (13.1)	20	9 (75)	2.3 (25.1)	7 months	20	8.11 (65)	1.2 (13.1)	20	7.47 (58)	2.3 (25.1)
5. Tildesley, H. D., 2010 [5]	23	8.5 (69)	1.2 (13.1)	24	8.8 (73)	1.3 (14.2)	6 months	23	8.4 (68)	1.4 (15.3)	24	7.6 (60)	0.74 (8.1)
6. Cho, J. H., 2006 [6]	40	7.5 (58)	1.3 (14.2)	40	7.7 (61)	1.5 (16.4)	30 months	36	7.4 (57)	1.3 (14.2)	35	6.7 (50)	0.9 (9.8)
7. Kwon, H. S., 2004 [7]	55	7.19 (55)	1.17 (12.8)	55	7.59 (59)	1.43 (15.6)	3 months	50	7.62 (60)	0.92 (10.1)	51	6.94 (52)	0.93 (10.2)
8. Rodriguez-Idigoras, M. I., 2009 [8]	167	7.41 (57)	1.32 (14.4)	161	7.62 (60)	1.62 (17.7)	12 months	151	7.35 (57)	1.32 (14.4)	146	7.4 (57)	1.39 (15.2)
9. Lim, S., 2016 [9]	50	7.9 (63)	0.8 (8.7)	50	8.1 (65)	0.9 (9.8)	6 months	42	7.9 (63)	1.2 (13.1)	43	7.3 (56)	0.9 (9.8)
10. Forjuoh, S. N., 2014 [10]	95	9.2 (77)	1.6 (17.5)	81	9.3 (78)	1.6 (17.5)	12 months	73	8.5 (69)	1.6 (17.5)	47	8.6 (70)	1.6 (17.5)
11. Glasgow, R. E., 2010 [11]	132	8.06 (65)	1.76 (19.2)	169	8.01 (64)	1.85 (20.2)	12 months	132	8.04 (64)	1.61 (17.6)	169	8.1 (65)	1.82 (19.9)
12. Quinn, C. C., 2011 [12]	56	9.2 (77)	1.7 (18.6)	22	9 (75)	1.8 (19.7)	12 months	51	8.5 (69)	1.8 (19.7)	21	7.9 (63)	1.4 (15.3)
12. Quinn, C. C., 2011 [12]	56	9.2 (77)	1.7 (18.6)	62	9.9 (85)	2.1 (23.0)	12 months	51	8.5 (69)	1.8 (19.7)	56	7.9 (63)	1.7 (18.6)
13. Bujnowska-Fedak, 2011 [13]	48	7.61 (60)	1.65 (18.0)	47	7.63 (60)	1.53 (16.7)	6 months	48	7.43 (58)	1.49 (16.3)	47	7.37 (57)	1.27 (13.9)
14. Hsu, W. C., 2016 [14]	20	10.9 (96)	1.2 (13.1)	20	10.8 (95)	1.2 (13.1)	12±2 weeks	20	8.9 (74)	2.2 (24.0)	20	7.7 (61)	1.6 (17.5)
15. Dario, C., 2016 [15]	91	7.93 (63)	1.1 (12.0)	208	7.94 (63)	0.98 (10.7)	12 months	78	7.66 (60)	1.1 (12.0)	168	7.68 (61)	0.98 (10.7)
16. Torbjornsen, A., 2014 [16]	50	8.3 (67)	1.2 (13.1)	51	8.1 (65)	1.1 (12.0)	12 months	41	8.2 (66)	1.33 (14.5)	39	7.8 (62)	1.03 (11.3)
17. Kardas, P., 2016 [17]	29	6.84 (51)	0.98 (10.7)	30	6.78 (51)	1.1 (12.0)	6 weeks	29	6.78 (51)	0.92 (10.1)	30	6.75 (50)	0.95 (10.4)
18. Nicolucci, A., 2015 [18]	149	8 (64)	0.8 (8.7)	153	7.9 (63)	0.7 (7.7)	12 months	135	7.78 (62)	1.1 (12.0)	114	7.44 (58)	1 (10.9)

19. Tang, P. C., 2013 [19]	213	9.28 (78)	1.74 (19)	202	9.24 (77)	1.59 (17.4)	12 months	193	8.33 (68)	1.81 (19.8)	186	8.1 (65)	1.68 (18.4)
20. Kim, H. S., 2007 [20]	26	7.59 (59)	1.09 (11.9)	25	8.09 (65)	1.72 (18.8)	6 months	26	7.7 (61)	0.9 (9.8)	25	7.04 (53)	1.39 (15.2)
21. Kim, C. S., 2010 [21]	45	9.8 (84)	1.2 (13.1)	47	9.8 (84)	1.3 (14.2)	3 months	45	7.8 (62)	0.8 (8.7)	47	7.4 (57)	0.7 (7.7)
22. Wakefield, B. J., 2014 [22]	53	7.4 (57)	1.31 (14.3)	41	7.2 (55)	1.34 (14.6)	3 months	53	7.4 (57)	1.31 (14.3)	41	7.2 (55)	1.28 (14.0)
23. Stone, R. A., 2010 [23]	73	9.4 (79)	1.4 (15.3)	64	9.6 (81)	1.6 (17.5)	6 months	73	8.6 (70)	1.3 (14.2)	64	7.9 (63)	1.2 (13.1)
24. Pressman, A. R., 2014 [24]	91	9.2 (77)	1.5 (16.4)	107	9.4 (79)	1.7 (18.6)	6 months	91	7.4 (57)	1.5 (16.4)	107	7.4 (57)	1.7 (18.6)
25. Steventon, A., 2014 [25]	191	8.41 (68)	1.64 (17.9)	266	8.38 (68)	1.68 (18.4)	12 months	191	8.38 (68)	1.6 (17.5)	266	8.15 (66)	1.49 (16.3)
26. Waki, K., MD, 2014 [26]	27	7 (53)	0.9 (9.8)	27	7.1 (54)	1 (10.9)	3 months	27	7.1 (54)	1.1 (12.0)	27	6.7 (50)	0.7 (7.7)
27. Greenwood, D. A., 2015 [27]	45	8.2 (66)	1.1 (12)	45	8.5 (69)	1.1 (12.0)	6 months	40	7.46 (58)	1.1 (12.0)	40	7.35 (57)	1.1 (12.0)
28. Weinstock, R. S., 2011 [28]	821	7.45 (58)	1.72 (18.8)	844	7.43 (58)	1.45 (15.8)	5 years	821	7.38 (57)	1.72 (18.8)	844	7.09 (54)	1.74 (19.0)
29. Liu, C.T., 2005	134	8.95 (74)	2.23 (24.4)	140	9.03 (75)	2.79 (30.5)	8 months	134	8.03 (64)	1.55 (16.9)	140	7.38 (57)	1.37 (15.0)
30. Kim, H.S., 2008	30	7.66 (60)	0.7 (7.7)	30	8.16 (66)	1.9 (20.8)	6 months	16	7.66 (60)	0.5 (5.5)	18	6.94 (52)	1.1 (12.0)
31. Faridi, Z., 2008	15	6.5 (48)	0.7 (7.7)	15	6.4 (46)	0.6 (6.6)	3 months	15	6.8 (51)	0.7 (7.7)	15	6.3 (45)	0.6 (6.6)
32. McMahon, G.T., 2005	52	9.9 (85)	0.8 (8.7)	52	10 (86)	0.8 (8.7)	12 months	52	8.7 (72)	0.8 (8.7)	52	8.4 (68)	0.8 (8.7)
33. Takenga, C., 2014	20	8.59 (70)	1.21 (13.2)	20	8.67 (71)	1.22 (13.3)	2 months	14	8.6 (70)	1.35 (14.8)	17	6.73 (50)	1.59 (17.4)
34. Yoo, H.J., 2009	54	7.4 (57)	0.9 (9.8)	57	7.6 (60)	0.9 (9.8)	3 months	54	7.6 (60)	1.0 (10.9)	57	7.1 (54)	0.8 (8.7)
35. Yoon, K.H., 2008	26	7.59 (59)	1.09 (11.9)	25	8.09 (65)	1.72 (18.8)	12 months	26	8.4 (68)	1.04 (11.4)	25	6.77 (50)	0.77 (8.4)

Reference

1. Zhou P, Xu L, Liu X, Huang J, Xu W, Chen W. Web-based telemedicine for management of type 2 diabetes through glucose uploads: a randomized controlled trial. Int J Clin Exp Pathol 2014;7:8848-8854. PMID: 25674254
2. Orsama AL, Lahteenmaki J, Harno K, Kulju M, Wintergerst E, Schachner H, Stenger P, Leppanen J, Kaijanranta H, Salaspuro V, Fisher WA. Active assistance technology reduces glycosylated hemoglobin and weight in individuals with type 2 diabetes: results of a theory-based randomized trial. Diabetes

Technol Ther 2013;15:662-669. PMID: 23844570
3. Avdal EU, Kizilci S, Demirel N. The effects of web-based diabetes education on diabetes care results: a randomized control study. Comput Inform Nurs 2011;29:101-106. PMID: 21099675
4. Noh JH, Cho YJ, Nam HW, Kim JH, Kim DJ, Yoo HS, Kwon YW, Woo MH, Cho JW, Hong MH, Yoo JH, Gu MJ, Kim SA, An KE, Jang SM, Kim EK, Yoo HJ. Web-based comprehensive information system for self-management of diabetes mellitus. Diabetes Technol Ther 2010;12:333-337. PMID: 20388042
5. Tildesley HD, Mazanderani AB, Ross SA. Effect of Internet therapeutic intervention on A1C levels in patients with type 2 diabetes treated with insulin. Diabetes Care 2010;33:1738-1740. PMID: 20668152
6. Cho JH, Chang SA, Kwon HS, Choi YH, Ko SH, Moon SD, Yoo SJ, Song KH, Son HS, Kim HS, Lee WC, Cha BY, Son HY, Yoon KH. Long-term effect of the Internet-based glucose monitoring system on HbA1c reduction and glucose stability: a 30-month follow-up study for diabetes management with a ubiquitous medical care system. Diabetes Care 2006;29:2625-2631. PMID: 17130195
7. Kwon HS, Cho JH, Kim HS, Song BR, Ko SH, Lee JM, Kim SR, Chang SA, Kim HS, Cha BY, Lee KW, Son HY, Lee JH, Lee WC, Yoon KH. Establishment of blood glucose monitoring system using the internet. Diabetes Care 2004;27:478-483. PMID: 14747232
8. Rodriguez-Idigoras MI, Sepulveda-Munoz J, Sanchez-Garrido-Escudero R, Martinez-Gonzalez JL, Escolar-Castello JL, Paniagua-Gomez IM, Bernal-Lopez R, Fuentes-Simon MV, Garofano-Serrano D. Telemedicine influence on the follow-up of type 2 diabetes patients. Diabetes Technol Ther 2009;11:431-437. PMID: 19580356
9. Lim S, Kang SM, Kim KM, Moon JH, Choi SH, Hwang H, Jung HS, Park KS, Ryu JO, Jang HC. Multifactorial intervention in diabetes care using real-time monitoring and tailored feedback in type 2 diabetes. Acta Diabetol 2016;53:189-198. PMID: 25936739
10. Forjuoh SN, Bolin JN, Huber JC, Jr., Vuong AM, Adepoju OE, Helduser JW, Begaye DS, Robertson A, Moudouni DM, Bonner TJ, McLeroy KR, Ory MG. Behavioral and technological interventions targeting glycemic control in a racially/ethnically diverse population: a randomized controlled trial. BMC Public Health 2014;14:71. PMID: 24450992
11. Glasgow RE, Kurz D, King D, Dickman JM, Faber AJ, Halterman E, Wooley T, Toobert DJ, Strycker LA, Estabrooks PA, Osuna D, Ritzwoller D. Outcomes of minimal and moderate support versions of an internet-based diabetes self-management support program. J Gen Intern Med 2010;25:1315-1322. PMID: 20714820
12. Quinn CC, Shardell MD, Terrin ML, Barr EA, Ballew SH, Gruber-Baldini AL. Cluster-randomized trial of a mobile phone personalized behavioral intervention for blood glucose control. Diabetes Care 2011;34:1934-1942. PMID: 21788632

13. Bujnowska-Fedak MM, Puchala E, Steciwko A. The impact of telehome care on health status and quality of life among patients with diabetes in a primary care setting in Poland. Telemed J E Health 2011;17:153-163. PMID: 21375410
14. Hsu WC, Lau KH, Huang R, Ghiloni S, Le H, Gilroy S, Abrahamson M, Moore J. Utilization of a Cloud-Based Diabetes Management Program for Insulin Initiation and Titration Enables Collaborative Decision Making Between Healthcare Providers and Patients. Diabetes Technol Ther 2016;18:59-67. PMID: 26645932
15. Dario C, Toffanin R, Calcaterra F, Saccavini C, Stafylas P, Mancin S, Vio E. Telemonitoring of Type 2 Diabetes Mellitus in Italy. Telemedicine journal and e-health : the official journal of the American Telemedicine Association 2016. PMID: 27379995
16. Torbjornsen A, Jenum AK, Smastuen MC, Arsand E, Holmen H, Wahl AK, Ribu L. A Low-Intensity Mobile Health Intervention With and Without Health Counseling for Persons With Type 2 Diabetes, Part 1: Baseline and Short-Term Results From a Randomized Controlled Trial in the Norwegian Part of RENEWING HEALTH. JMIR Mhealth Uhealth 2014;2:e52. PMID: 25499592
17. Kardas P, Lewandowski K, Bromuri S. Type 2 Diabetes Patients Benefit from the COMODITY12 mHealth System: Results of a Randomised Trial. J Med Syst 2016;40:259. PMID: 27722974
18. Nicolucci A, Cercone S, Chiriatti A, Muscas F, Gensini G. A Randomized Trial on Home Telemonitoring for the Management of Metabolic and Cardiovascular Risk in Patients with Type 2 Diabetes. Diabetes Technol Ther 2015;17:563-570. PMID: 26154338
19. Tang PC, Overhage JM, Chan AS, Brown NL, Aghighi B, Entwistle MP, Hui SL, Hyde SM, Klieman LH, Mitchell CJ, Perkins AJ, Qureshi LS, Waltimyer TA, Winters LJ, Young CY. Online disease management of diabetes: engaging and motivating patients online with enhanced resources-diabetes (EMPOWER-D), a randomized controlled trial. J Am Med Inform Assoc 2013;20:526-534. PMID: 23171659
20. Kim HS, Jeong HS. A nurse short message service by cellular phone in type-2 diabetic patients for six months. J Clin Nurs 2007;16:1082-1087. PMID: 17518883
21. Kim CS, Park SY, Kang JG, Lee SJ, Ihm SH, Choi MG, Yoo HJ. Insulin dose titration system in diabetes patients using a short messaging service automatically produced by a knowledge matrix. Diabetes Technol Ther 2010;12:663-669. PMID: 20615108
22. Wakefield BJ, Koopman RJ, Keplinger LE, Bomar M, Bernt B, Johanning JL, Kruse RL, Davis JW, Wakefield DS, Mehr DR. Effect of home telemonitoring on glycemic and blood pressure control in primary care clinic patients with diabetes. Telemed J E Health 2014;20:199-205. PMID: 24404819
23. Stone RA, Rao RH, Sevick MA, Cheng C, Hough LJ, Macpherson DS, Franko CM, Anglin RA, Obrosky DS, Derubertis FR. Active care management supported by home telemonitoring in veterans with type 2 diabetes: the DiaTel randomized controlled trial. Diabetes Care 2010;33:478-484. PMID: 20009091

24. Pressman AR, Kinoshita L, Kirk S, Barbosa GM, Chou C, Minkoff J. A novel telemonitoring device for improving diabetes control: protocol and results from a randomized clinical trial. Telemed J E Health 2014;20:109-114. PMID: 24404816

25. Steventon A, Bardsley M, Doll H, Tuckey E, Newman SP. Effect of telehealth on glycaemic control: analysis of patients with type 2 diabetes in the Whole Systems Demonstrator cluster randomised trial. BMC Health Serv Res 2014;14:334. PMID: 25100190

26. Waki K, Fujita H, Uchimura Y, Omae K, Aramaki E, Kato S, Lee H, Kobayashi H, Kadowaki T, Ohe K. DialBetics: A Novel Smartphone-based Self-management Support System for Type 2 Diabetes Patients. Journal of Diabetes Science & Technology 2014;8:209. PMID: 24876569

27. Greenwood DA, Blozis SA, Young HM, Nesbitt TS, Quinn CC. Overcoming Clinical Inertia: A Randomized Clinical Trial of a Telehealth Remote Monitoring Intervention Using Paired Glucose Testing in Adults With Type 2 Diabetes. J Med Internet Res 2015;17:e178. PMID: 26199142

28. Weinstock RS, Teresi JA, Goland R, Izquierdo R, Palmas W, Eimicke JP, Ebner S, Shea S. Glycemic control and health disparities in older ethnically diverse underserved adults with diabetes: five-year results from the Informatics for Diabetes Education and Telemedicine (IDEATel) study. Diabetes Care 2011;34:274-279. PMID: 21270184

29. Liu CT YY, Lee TI, Li YC. Observations on online services for diabetes management. Diabetes Care 2005;28:2807-08:1-7. PMID: 16249564

30. Kim H-S, Song M-S. Technological intervention for obese patients with type 2 diabetes. Applied Nursing Research 2008;21:84-89. PMID: 18457747

31. Faridi Z, Liberti L, Shuval K, Northrup V, Ali A, Katz DL. Evaluating the impact of mobile telephone technology on type 2 diabetic patients' self-management: the NICHE pilot study. Journal of Evaluation in Clinical Practice 2008;14:465-469. PMID: 18373577

32. McMahon GT, Gomes HE, Hickson Hohne S, Hu TM-J, Levine BA, Conlin PR. Web-based care management in patients with poorly controlled diabetes. Diabetes care 2005;28:1624-1629. PMID: 15983311

33. Takenga C, Berndt R-D, Musongya O, Kitero J, Katoke R, Molo K, Kazingufu B, Meni M, Vikandy M, Takenga H. An ICT-Based Diabetes Management System Tested for Health Care Delivery in the African Context. International Journal of Telemedicine and Applications 2014;2014:437307-437310. PMID: 25136358

34. Yoo HJ, Park MS, Kim TN, Yang SJ, Cho GJ, Hwang TG, Baik SH, Choi DS, Park GH, Choi KM. A Ubiquitous Chronic Disease Care system using cellular phones and the internet. Diabetic medicine : a journal of the British Diabetic Association 2009;26:628-635. PMID: 19538239

35. Yoon K-H, Kim H-S. A short message service by cellular phone in type 2 diabetic patients for 12 months. Diabetes research and clinical practice 2008;79:256-261. PMID: 17988756
